# Geometric morphometric analysis of maxillary molar sexual dimorphism: A cross-sectional study

**DOI:** 10.1371/journal.pone.0355612

**Published:** 2026-08-07

**Authors:** Srikant Natarajan, Junaid Ahmed, Shravan Shetty, Nidhin Philip Jose, Sharada Chowdappa, Sunitha Carnelio

**Affiliations:** 1 Department of Oral Pathology and Microbiology, Manipal College of Dental Sciences Mangalore, Manipal Academy of Higher Education, Manipal, Karnataka, India; 2 Department of Oral Medicine and Radiology, Manipal College of Dental Sciences Mangalore, Manipal Academy of Higher Education, Manipal, Karnataka, India; 3 Department of Orthodontics and Dentofacial Orthopaedics, Manipal College of Dental Sciences Mangalore, Manipal Academy of Higher Education, Manipal, Karnataka, India; 4 Private Dental Researcher, Mangaluru, Karnataka, India; 5 Department of Oral Pathology and Microbiology, Manipal College of Dental Sciences, Manipal Academy of Higher Education, Manipal, Karnataka, India; Danube Private University, AUSTRIA

## Abstract

**Objective:**

The sexual dimorphism of permanent maxillary molars has been extensively documented through metric and subjective shape analysis. The aim of this study was to evaluate sexual dimorphism in the shape of permanent maxillary first (UM1) and second molars (UM2) using geometric morphometric analysis.

**Methods:**

This cross-sectional study analysed digitized dental casts from 60 males and 60 females. The casts were scanned using a laser surface scanner, and 28 three-dimensional landmarks, defined based on anatomical and geometric landmarks, were marked using SlicerMorph software. The shape data was then analysed using Procrustes superimposition, principal components analysis, regression, and discriminant function analysis to test the shape changes, allometry, and differences between the sexes.

**Results:**

Centroid size did not differ significantly between sexes for UM1 and UM2 (p > 0.05). Shape differences were not significant in UM1 (p = 0.15) but were significant in UM2 (p = 0.01). Allometry was observed in both molars, with a greater contribution in UM2 **(**6.96%**)** compared to UM1 **(**2.39%**)** (p < 0.001). Discriminant function analysis identified patterns of shape variation between groups; however, these findings should be interpreted cautiously as no cross-validation was performed.

**Conclusion:**

Geometric morphometric analysis identified significant shape differences in the maxillary second molar. These findings demonstrate the utility of 3D geometric morphometric analysis for assessing tooth shape variation and support its application to evaluating sexual dimorphism of maxillary molars. Further studies involving larger and more diverse populations are warranted to validate these findings.

## Introduction

Advances in three-dimensional digital imaging have facilitated detailed evaluation of dental morphology and enabled quantitative assessment of tooth shape using geometric morphometric methods. Human molars exhibit considerable morphological variation, making them a useful model for investigating patterns of dental shape variation and sexual dimorphism [1]. The modern human tooth phenotype has evolved over time, with features being gained or lost. These changes are most easily observed in human molars [2]. An example is the Cope Osborne theory, which explains the reduction of the distopalatal cusp size and associated translation in contour from the rhomboidal permanent first molar to the heart-shaped second and third molars. Another important trait is the cusp of Carabelli, which is typically absent in the second and third molars and missing in a small percentage of first molars as well [3,4].

Tooth size is influenced by a variety of epigenetic factors, including nutrition and hormonal status, maternal aspects, childhood health in addition to genetic factors [5–7]. Variations in overall body size may influence tooth dimensions, such that individuals with a larger build may exhibit larger teeth irrespective of sex. For instance, a larger-built female may have a molar dimension comparable to or exceeding that of a smaller-built male, limiting the reliability of size as an indicator of sexual dimorphism [5–7]. In contrast, tooth shape is considered to be more strongly influenced by genetic factors and is relatively less affected by such variations [8]. Therefore, evaluating sexual dimorphism through landmark-based geometric analysis of tooth morphology may provide a more consistent and reliable approach than conventional size-based assessments.

The development of teeth is a complex process involving interactions between the ectoderm and ectomesenchyme [9]. During tooth development, the inner enamel epithelium (ectoderm-derived) interacts with the dental papilla (ectomesenchyme-derived) through reciprocal epithelial–mesenchymal signaling, ultimately leading to the formation of dental hard tissues. [9]. The enamel–dentin junction is largely determined by genetic factors during tooth development [9]. Sex-linked differences in amelogenin expression and chromosomal composition have been associated with variation in enamel and dentin thickness, providing a biological basis for studying dental sexual dimorphism [10,11].

Recent research on odontometry has shown that while males and females often have overlapping linear dimensions, males tend to have slightly larger dimensions. Enamel thickness is associated with the X chromosome, while the presence of the Y chromosome has been reported to influence both enamel and dentin dimensions. However, dentin formation also occurs in females and is not dependent on the presence of the Y chromosome [9,10]. Assessment of sexual dimorphism in tooth morphology has also been explored in forensic contexts, particularly for sex estimation when other biological indicators are unavailable [12,13]. However, the present study focuses on evaluating shape variation using geometric morphometric methods rather than direct forensic application.

Maxillary molars were specifically selected for this study due to their unique occlusal morphology and well-documented cusp and groove patterns, particularly the hypocone and cusp of Carabelli, which are known to exhibit significant variation [14]. Additionally, the first and second maxillary molars demonstrate comparable anatomical landmarks and functional characteristics, allowing for consistent landmark placement and reliable shape comparison, unlike mandibular molars, which exhibit greater morphological variability. Maxillary molars have also been widely used in morphometric and anthropological studies due to their diagnostic relevance, making them a suitable model for evaluating shape-based sexual dimorphism using geometric morphometric approaches [15].

Currently, the majority of the research focuses on size-related measurements rather than the shape of biological entities. Understanding sexual dimorphism in molars provides a quantitative framework for evaluating dental shape and morphological variation beyond conventional linear measurements. Such an approach contributes to the quantitative assessment of tooth morphology and provides a framework for future morphometric and comparative dental studies.

This study was undertaken to evaluate sexual dimorphism in the shape and size of permanent maxillary first (UM1) and second molars (UM2) using three-dimensional geometric morphometric analysis. The null hypothesis was that there is no significant difference in the shape or size of maxillary permanent molars between males and females.

## Materials and methods

### Ethical approval and consent to participate

All procedures performed in this study involving human participants were conducted in accordance with the ethical standards of the institutional research committee and with the 1964 Declaration of Helsinki and its later amendments, or comparable ethical standards [16]. Approval for the study was obtained from the institutional ethics committee (reference number 20018, dated 16th March 2020) prior to the commencement of the study. This observational cross-sectional study was not prospectively preregistered, as preregistration was not a routine institutional requirement for observational studies at the time the study was initiated. All data were anonymized, and written informed consent for research use was obtained from participants or their legal guardians, as applicable.

The pretreatment dental casts used in this study formed part of the routine orthodontic records obtained during clinical care. The inclusion of younger participants was based on the presence of fully erupted permanent maxillary molars, as crown morphology is complete at this stage and not influenced by subsequent growth. The casts were part of routine clinical records, and no additional procedures were performed for research purposes.

Detailed demographic information, including the birthplace and domicile of the participant, parents, and grandparents, was recorded. An individual was considered to belong to the Dakshina Kannada region if the participant, parents, and grandparents were born and had resided in the region. This criterion was adopted to reduce potential variability arising from geographic, environmental, and population-level differences within the study sample. However, complete population homogeneity cannot be assumed. Individuals with intact maxillary posterior teeth and no history of dental caries, trauma, or restorations were included in the study. Individuals with craniofacial anomalies, syndromes, or systemic conditions known to affect tooth development were excluded. Participants were selected using convenience sampling from patients meeting the predefined inclusion criteria during the study period.

### Sample size calculation

Sample size estimation was based on previously reported data on sexual dimorphism in mesiodistal dimensions of maxillary molars by Abe K et al. (1996) [17]. The reported mean ± standard deviation values of mesiodistal dimensions for males were 10.57 ± 0.52 mm (first molar) and 9.98 ± 0.58 mm (second molar), and for females were 10.15 ± 0.55 mm (first molar) and 9.47 ± 0.60 mm (second molar). These values were used to calculate the required sample size for comparison of means between two groups using the formula for calculation of sample size using two means,


$$\begin{array}{c} \text{N}=\text{2}[[{\left({\text{Z}}_{\text{1}-\frac{\alpha }{\text{2}}}+{\text{Z}}_{\text{1}-\beta }\right)}^{\text{2}}]\;\sigma^{\text{2}}]\;/\;{\text{d}}^{\text{2}} \end{array}$$ (1)

where d=clinically significant difference; σ=mean standard deviation. Using an alpha error of 1% (Z = 2.576), power of 90% (Z = 1.282), an average standard deviation of 0.535, and a clinically significant difference of 0.4 mm, the required sample size in each group was calculated as 54. The sample size was increased to 60 participants per group to accommodate potential variability and provide greater precision in the morphometric analyses, resulting in a total sample of 120 individuals. As no established sample size estimation approach was available for the specific three-dimensional geometric morphometric analyses employed in this study, previously reported linear odontometric measurements were used as a pragmatic guide for sample size determination. The final sample exceeded the number of variables included in the analysis, a condition generally regarded as favorable for multivariate statistical estimation.

**Fig 1:**
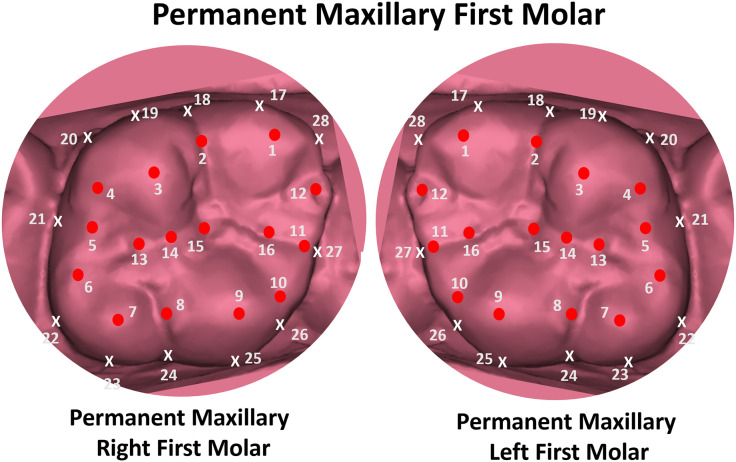
Landmark configuration of the permanent maxillary first molar. A total of 28 three-dimensional landmarks are shown, including anatomically defined landmarks (cusps, ridges, grooves, and pits) and geometrically defined landmarks (crests of curvature and line angles), as detailed in Table 1.

**Fig 2:**
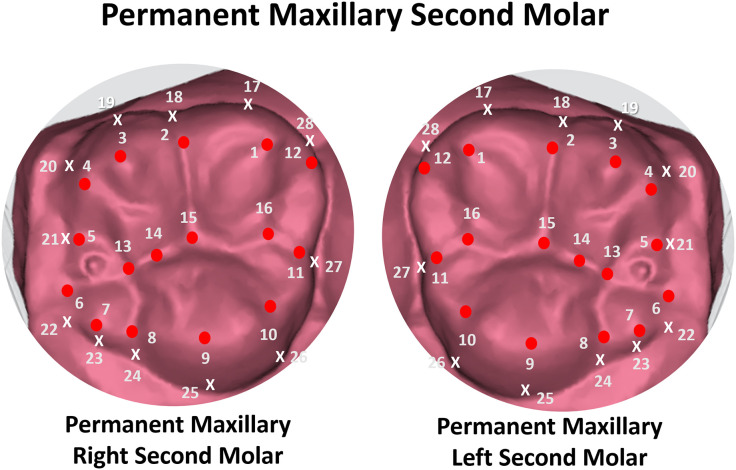
Landmark configuration of the permanent maxillary second molar. The same set of 28 three-dimensional anatomical and geometric landmarks as described for the first molar are illustrated to enable consistent shape comparison between molars.

In accordance with ethical research practices of the institutional ethics committee, the casts were obtained with the informed consent of the individual, which was properly documented through a signed agreement as a part of the treatment protocol of the department of orthodontics. The casts obtained following impression were digitized using laser surface scanner (inEOS X5; Densply Sirona, New Delhi, India). Twenty-eight landmarks were defined according to anatomic and geometric evidence in line with the proposal given by Biggerstaff R (1969) to describe the basal area of posterior teeth [18] and modified from the landmarks suggested by Robinson DL et al (2002) [19] and Al-Shahrani I et al (2014) [20]. We marked 18 landmarks based on “Anatomic evidence” (corresponding to cusp tips, fissure junctions, endpoints of common clinical measurements like mesiodistal width, buccolingual width, line angles, and point angles) and 10 landmarks based on “Geometric evidence” (based on the crests of curvature, line and point angles, surface landmarks corresponding to the occlusal surface landmarks) using three-dimensional morphometric software (SlicerMorph; open-source platform, Seattle, WA, USA; accessed from https://github.com/SlicerMorph) [21](Table 1, Figs 1 and 2). These landmarks provided a standardized set of reference points for quantifying three-dimensional variation in maxillary molar morphology.

**Table 1:** Anatomical and geometric landmarks used for three-dimensional geometric morphometric analysis of permanent maxillary molars, including cusp, ridge, groove, pit, and crest of curvature landmarks.

Landmark Number	Landmark description	Definition
Landmarks based on cusps (Anatomic Landmarks)		
Landmark 1	Mesiobuccal cusp tip	Junction of the mesial and distal slopes of the mesiobuccal cusp and triangular ridge of the mesiobuccal cusp
Landmark 3	Distobuccal cusp tip	Junction of the mesial and distal slopes of the distobuccal cusp and triangular ridge of the distobuccal cusp
Landmark 7	Distopalatal cusp tip	Junction of the mesial and distal slopes of the distopalatal cusp and triangular ridge of distopalatal cusp.
Landmark 9	Mesiopalatal cusp tip	Junction of the mesial and distal slopes of mesiopalatal cusp and triangular ridge(s) of mesiopalatal cusp
Landmarks based on ridges (Anatomic Landmarks)		
Landmark 4	Buccal end of the distal marginal ridge	Junction of the distal slope of distobuccal cusp and the distal marginal ridge.
Landmark 5	Midpoint of the distal marginal ridge	Midpoint of the distal marginal ridge on the occlusal surface
Landmark 6	Palatal end of the distal marginal ridge	Junction of the distal slope of distopalatal cusp and the distal marginal ridge
Landmark 10	Palatal end of the mesial marginal ridge	Junction of mesial slope of mesiopalatal cusp and the mesial marginal ridge.
Landmark 11	Midpoint of the mesial marginal ridge	Midpoint of the mesial marginal ridge on the occlusal surface
Landmark 12	Buccal end of the mesial marginal ridge	Junction of mesial cusp slope of mesiobuccal cusp and mesial marginal ridge
Landmarks based on grooves, pits and fissures (Anatomic Landmarks)		
Landmark 2	Buccal groove point	Junction of the distal slope of mesiobuccal cusp, mesial slope of distobuccal cusp and the buccal groove
Landmark 8	Palatal groove point	Junction of the distal slope of mesiopalatal cusp, mesial slope of distopalatal cusp and palatal groove
Landmark 13	Distal triangular pit	Junction of the central groove and the distal triangular fossa
Landmark 14	Groove of the oblique ridge	The midpoint of the groove crossing the oblique ridge
Landmark 15	Central pit	Meeting point of the buccal groove, Central Groove and the groove of the oblique ridge
Landmark 16	Mesial triangular pit	Junction of the central groove and the mesial triangular fossa
Landmark 18	Termination of the buccal groove	Termination of the buccal groove on the buccal surface
Landmark 24	Termination of the palatal groove	Termination of the palatal groove on the palatal surface
Landmarks based on crests of curvature (Geometric Landmarks)		
Landmark 17	Buccal crest of curvature on mesiobuccal lobe	Buccal crest of curvature on mesiobuccal lobe
Landmark 19	Buccal crest of curvature on the distobuccal lobe	Buccal crest of curvature on the distobuccal lobe
Landmark 20	Distobuccal line angle	The most occlusal crest of curvature of the junction of the distal and buccal surfaces
Landmark 21	Distal crest of curvature	distal most point on the distal surface
Landmark 22	Distopalatal line angle	The most occlusal crest of curvature of the junction of the distal and palatal surfaces
Landmark 23	Palatal crest of curvature on the distopalatal lobe	Palatal crest of curvature on the distopalatal lobe
Landmark 25	Palatal crest of curvature of distopalatal lobe	Palatal crest of curvature corresponding to the tip of the distopalatal cusp / cusp of carabelli if present
Landmark 26	Mesiopalatal line angle	The most occlusal crest of curvature of the junction of the mesial and palatal surfaces
Landmark 27	Mesial crest of curvature	Mesial most point on the contour of the mesial surface
Landmark 28	Mesiobuccal line angle	The most occlusal crest of curvature of the junction of the mesial and buccal surfaces

Note: The landmarks shown in Figs 1 and 2 are labelled with numbers corresponding to the landmark descriptions provided in the table.

The fiducial point marking tool of the morphometry software was utilized to accurately mark the landmarks. To ensure precise visualization, the casts were rotated or translated as needed. After marking the fiducial points with precision, the landmark coordinates were obtained in x, y, and z format. The extracted landmark coordinates were exported to spreadsheet software (Microsoft Excel; Microsoft Corporation, Redmond, WA, USA), with the x, y, and z coordinate values of each landmark arranged in horizontal sequence for subsequent analysis. This formatted file was subsequently analysed using geometric morphometric software (MorphoJ; Klingenberg CP, Manchester, UK; https://morphometrics.uk/MorphoJ_page.html) for shape analysis [22]. Both right and left maxillary molars were included in the analysis. Prior to averaging, landmark configurations from the left side were reflected (mirrored) into the orientation of the corresponding right-side molars to ensure homologous landmark correspondence. Landmark coordinates from both sides were then averaged for each individual to minimize the influence of side-related variation and generate a single representative configuration for subsequent analyses. As the primary objective of the study was to evaluate sexual dimorphism, bilateral asymmetry was not analysed separately. The unit of analysis in this study was the individual, with averaged landmark coordinates from bilateral molars used to represent each participant.

All observers underwent calibration and training in landmark identification prior to data collection to ensure consistency in landmark placement. Reliability of landmark identification was assessed using a random subsample of 20 dental models. For inter-observer reliability, two calibrated observers independently digitized the complete landmark configuration on the selected models. For intra-observer reliability, one observer repeated landmark placement on the same models after a five-day interval. Reliability was quantified using global intraclass correlation coefficients (ICC) calculated from the complete landmark coordinate datasets obtained during repeated digitization. The ICC values ranged from 0.982 to 1.000, indicating excellent agreement.

In addition, intra-operator reproducibility during the digitization stage was evaluated using repeated landmark digitization of the same subset of models. Measurement error was assessed in MorphoJ software using a one-way nonparametric ANOVA with a randomized permutation procedure (10,000 iterations), which partitioned the total variance into biological variation and digitization-error components. The variance attributable to digitization error for centroid size and shape was less than 10% of the total variance, indicating that measurement error was negligible relative to biological variation.

Statistical analysis was performed using geometric morphometric software (MorphoJ; Klingenberg CP, Manchester, UK). Geometric morphometric analysis was performed following Procrustes superimposition to remove the effects of size, position, and orientation, allowing comparison of shape variables. All statistical analyses were performed under standard assumptions of multivariate morphometric analysis following Procrustes superimposition. Principal components analysis (PCA) was used to explore patterns of shape variation and assess distribution between sexes. Procrustes ANOVA was employed to evaluate statistically significant differences in shape between groups. Regression analysis was performed to evaluate allometric relationships between size and shape, and was not intended for predictive classification. Discriminant function analysis (DFA) was used to evaluate the ability of shape variables to classify individuals by sex, and classification accuracy was calculated based on the original dataset. No cross-validation was performed, and results were interpreted with caution. Given the multivariate nature of shape data, classification was evaluated using discriminant function analysis rather than logistic regression-based approaches. The level of statistical significance was set at p < 0.05.

## Results

The study included 120 participants (60 males and 60 females), with an overall mean age of 18.6 years (SD = 2.5) with females having a mean age of 17.9 years (SD = 2.8) and males 19.5 years (SD = 1.7). The landmarks were obtained from the 120 digitized casts (constituting 60 males and 60 females), of individuals from the Dakshina Kannada, Mangaluru, region of Karnataka, India. Procrustes ANOVA analysis showed that the centroid scores accounted for 4.17% and 6.74% of the variation between the sexes in the permanent maxillary first and second molars, respectively and were not statistically significant. The shape data showed significant variation between the sexes in the maxillary second molar (*p =* 0.010) but not in the first molar (*p =* 0.150) (Table 2).

**Table 2:** Procrustes ANOVA comparing centroid size and shape between sexes in permanent maxillary first and second molars.

	Maxillary First Molar		Maxillary Second Molar	
	Centroid	Shape	Centroid	Shape
Sum of Squares	4.178	0.0157	6.74	0.0241
Mean Sum of Squares	4.178	0.000205	6.74	0.000313
F	3.38	1.17	1.77	1.41
*p*	0.0686	0.150	0.1865	0.010

Regression analysis demonstrated a significant association between size and shape in both the first and second molars, indicating the presence of allometry. Both the first and second molars demonstrated significant allometric effects, with centroid size explaining 2.39% and 6.96% of shape variation, respectively (p < 0.001) (Table 3).

**Table 3:** Regression Analysis showing the relationship between centroid size and shape (allometry) in permanent maxillary molars.

	Maxillary First Molar	Maxillary Second Molar
Total SS:	1.608525	2.037685
Predicted SS:	0.038448	0.141728
Residual SS:	1.570077	1.895957
% predicted: (Allometry)	2.39%	6.96%
*p*	0.001	< 0.001

SS: Sum of Squares.

Procrustes distance quantifies the magnitude of shape difference between mean configurations, whereas Mahalanobis distance measures multivariate separation between groups based on shape variables [3]. Discriminant function analysis identified patterns of shape variation between the sexes. Although the apparent in-sample classification accuracy was high, these findings should be interpreted cautiously because no cross-validation or external validation was performed. The Procrustes distances, representing the magnitude of shape difference between male and female mean configurations, were 0.0229 and 0.0284, the Mahalanobis distances were 3.294 and 3.0694 in the permanent maxillary first and second molars respectively. The prediction accuracy in females was higher at 96.67% in maxillary first molar and in males (96.67%) for maxillary second molar (Table 4). Although classification accuracy was high for both molars, statistically significant shape differences between sexes were observed only in the second molar. The classification accuracies reported represent apparent in-sample performance and should be interpreted cautiously, as no cross-validation or external validation was performed (Table 4).

**Table 4:** Exploratory discriminant function analysis of shape differences between sexes in permanent maxillary molars (classification results represent apparent in-sample performance and were not cross-validated).

	Permanent Maxillary First Molar	Permanent Maxillary Second Molar
Procrustes distance	0.0229	0.0284
Mahalanobis distance	3.2940	3.0694
T-square	325.51	282.64
*p*	0.075	0.128
Prediction accuracy Female	96.67	93.33
Prediction accuracy Male	95.00	96.67
Overall accuracy	95.83	95.00

**Fig 3:**
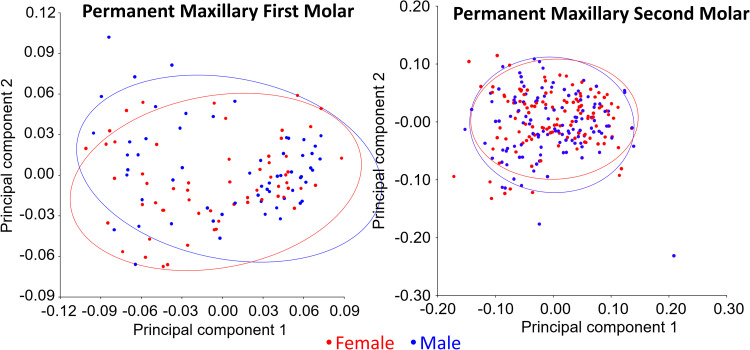
Principal component analysis (PCA) of shape variables in permanent maxillary molars. The distribution of samples along PC1 and PC2 demonstrates overall variation in shape, with substantial overlap between male and female groups, indicating limited separation.

Principal components analysis of the landmark data of maxillary first and second molar shows that the first 20/77 components account for 80% of the variance. The scatterplot of the PC1 and PC2 indicates a homogeneous distribution of samples in both sexes (Fig 3). Although high classification accuracy was observed, the associated group differences were not statistically significant, and principal component analysis demonstrated overlap between sexes. Therefore, the classification results should be interpreted with caution.

**Fig 4:**
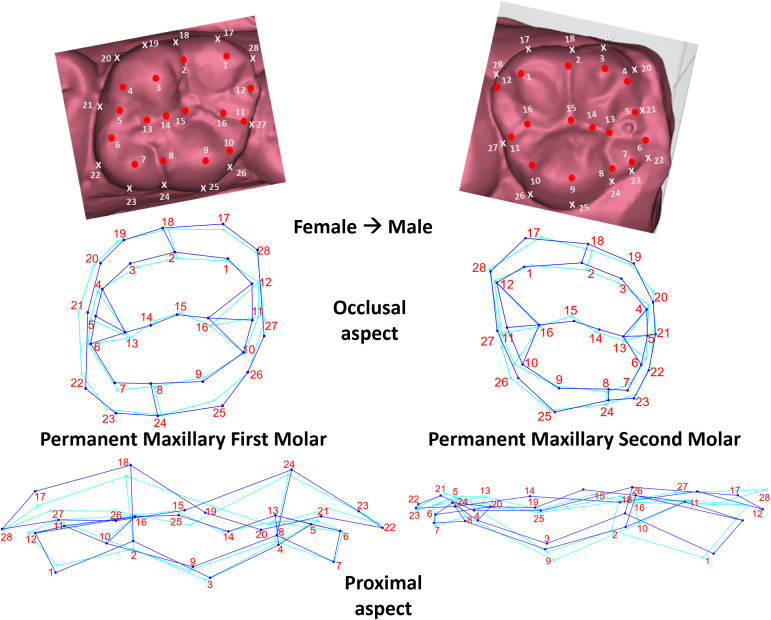
Relative landmark displacement between males and females derived from discriminant function analysis. The average female configuration is represented by the light blue outline and the average male configuration by the dark blue outline. Superimposition of the landmark configurations illustrates the relative displacement of landmarks contributing to observed shape variation. The figure is intended to visualize morphological differences and should not be interpreted as evidence of classification performance.

Discriminant function analysis of the landmarks of the permanent maxillary first molar showed sex-related differences in landmark configurations. The mesial outline was relatively more laterally positioned in females compared to males, the prominence of the crest of curvature of the mesiopalatal cusp (cusp of carabeli contour) is more in males (landmark 25). The distal marginal ridge region (landmarks 11, 27) in females and the mesial marginal ridge region (landmarks 5, 21) in males exhibited relative closer positioning of landmarks towards each other, reflecting localized differences in shape rather than direct linear narrowing. Another finding noted was that the occlusal table was more buccally positioned in males compared to females (landmarks 1, 2, 3) and the mesial and distal pit were relatively more palatally positioned in females (landmarks 13, 16). In the vertical dimension, males had more cervically positioned buccal crest of contour (landmark 17), and deeper buccal groove (landmark 18) (Fig 4).

Discriminant function analysis of the landmarks of permanent maxillary second molar shows that, the central pit was more buccally positioned in females (landmark 15), the occlusal table landmarks were more buccally positioned in males (landmark 2, 3). The distobuccal cusp tip was more buccally oriented in males (landmark 3). Female teeth exhibit more rounded contour in relation to the mesiopalatal line angle (landmarks 25, 26, 27). The mesial and distal marginal ridge regions (landmarks 5, 21 and 11, 27) in females showed a relative inward orientation compared to males, indicating localized shape variation. The crest of curvature of the mesiobuccal cusp (landmark 17) was relatively more mesially positioned in males. In the vertical dimension, females demonstrated relatively greater mesiobuccal and mesiopalatal cusp heights (landmarks 1, 9). Additionally, female teeth demonstrated a relatively more cervical position of the mesiobuccal line angle crest (landmark 28) and the buccal end of the mesial marginal ridge (landmark 12) (Fig 4).

## Discussion

In the present study, we evaluated sexual dimorphism in the shape of the permanent maxillary first (UM1) and second molars (UM2) using three-dimensional geometric morphometric analysis. The present study demonstrated a significant difference in the shape of the maxillary second molar between the sexes, whereas no significant difference was observed for the first molar.

Butler’s field model (1939) and Osborn’s Clone model (1978) are the two theories that describe tooth formation [11,23,24]. The clone model regards the shape of the teeth as self-generated, whereas the field model correlates the shape with a gradient of the mesenchymal expression. The permanent molars of modern humans are distal extensions of the field of the deciduous molar. Thus, as they get farther from the primary field (i.e., the region of the deciduous second molar), the characteristics resembling the deciduous second molar diminish. This is seen as the reduction in the hypocone structure in permanent maxillary second molars in our sample [25]. A study by Batbayar et al. (2016), reported that in the absence of the maxillary first molar, the second molar size exhibited increased crown size compared to the individuals with the presence of first molar [26]. This indicates that the second molar coming “closer” to the field of first molar resembles the first molar in size and shape; in contrast, the farther the second molar is, it will show higher variation in shape. This principle is supported by our results, where the maxillary second molar showed significant differences in shape between sexes (*p =* 0.01) as compared to first molar (*p =* 0.15), probably due to its more distant location from the primary field of influence. The significant difference in the shape of the second molar is predominantly attributed due to variation in the extent of hypocone (distopalatal cusp) development. The distopalatal region of the molar exhibits the greatest shape variation, which corresponds to the hypocone; this is consistent with the findings of Morita et al. (2014), who reported higher variability in the hypocone compared to other cusps of the maxillary second molar [9]. Their developmental analysis showed that crown size is dependent on the mesenchymal tissue possibly influencing the formation of the secondary enamel knots. They also reported that prolonged cusp development may occur at the expense of later-forming cusps, resulting in increased variability in these cusps—particularly the hypocone (distopalatal cusp) of the maxillary second molar. In contrast, such variability was not observed in the maxillary first molar, with greater variation consistently noted in later-forming cusps [9]. These findings support the observation that maxillary first molar has a more stable shape than second molar explaining the lack of significant difference in our study.

The increased variation of the distal molar can be explained by the shape variation, i.e., tooth morphological integration observed under the influence of the functional and/or developmental factors. There is a certain degree of modularity (which refers to the degrees of connectivity in systems like functional, developmental, and / or genetic factors influencing formation of a biological structure) observed in the formation of jaws and teeth. In relation to the tooth formation, the determination of the shape of the tooth is modulated by the functional constraints noted in relation to the space available, the formation of the cranium and the face and other factors [8]. These functional constraints prevent the tooth from undergoing directional or random changes as the cusps of the occluding teeth must fit optimally to maintain occlusion and thereby providing a stable masticatory function [27]. The first molar is a key determinant of occlusion because it erupts early and establishes the vertical and transverse dimensions of the dental arch as demonstrated by Angle’s classic work in 1899 [28]. Its shape, particularly the occlusal morphology and cusp positioning, contributes to the development of occlusal relationships, including intercuspation and arch coordination. Variations in the first molar’s shape (e.g., reduced cusp height, asymmetry, or abnormal crown morphology) can possibly lead to deviations in occlusal development, affecting arch form, curve of Spee, and overall occlusal stability.

The shape of the molar has been correlated with craniofacial complexes by Polychronis and Halazonetis (2014) [29]. They found lack of significant shape covariation between craniofacial complex and the maxillary first molars. The authors attributed this to the partial genetic independence that exists during the development of molars. Despite common developmental pathways, each tooth may be regarded as a genetically independent variable [29]. It may be noted that although the cranial skeleton exhibits sexual dimorphism, evidence for such dimorphism in molars is inconsistent. A geometric morphometric study found no significant sexual dimorphism in the maxillary first and second molars in samples from Granada (Spain), which is similar to our results for the first molar [30].

The outer enamel shape of the tooth is known to correlate with the enamel dentinal junction, which is determined by the membrana preformitiva during tooth formation. This shape variation is observed again more in relation to the maxillary second molar than the first molar. A geometric morphometry study demonstrated sexual dimorphism in the shape of the upper maxillary second molar in a Romanian population [31]. The period of formation of the tooth, the thickness of enamel and rate of formation of enamel, influence the outer enamel shape of the crown. Enamel thickness is known to be different in the paracone (mesiobuccal cusp) and protocone (mesiopalatal cusp) in maxillary second molar compared to the first molar, and do not show similar direction of change in shape with development. This difference between the two teeth and the more distant location from the mesenchymal field of influence explains the presence of sexual dimorphism in second molar and not in first molar [9].

Allometry is the size dependent shape change noted in the biological structure. In our results, we found that the permanent maxillary second molar showed 6.96% variation of shape with size compared to only 2.39% variation in permanent maxillary first molar. This is consistent with a previous morphometric study that reported metameric variation in human molars [32]. Their study showed that the permanent upper second molar showed considerable allometry with size variation and enamel dentin junction morphologies. With increasing size of the second molar they appear similar to the first molar, indicating a tendency of all the forming maxillary molars to resemble the first molar [32]. In our previous report, we observed similar findings in the mandibular postcanine dentition, where the more posteriorly positioned teeth exhibited a higher degree of variation [33].

The findings presented in this manuscript were also discussed at the 19th National Conference of The Indian Association of Forensic Odontology, conducted by G Pulla Reddy Dental College and Hospital, Kurnool, Andhra Pradesh [34]. The discussions reinforced the multifactorial nature of molar shape variation, including the influence of space availability, palatal-side-dominated growth patterns, and surrounding developmental tissues. We must thus remain aware of the variation before using the discriminating power of shape of the tooth. The permanent maxillary first molar forms in utero and develops in a controlled environment. This may lead to less sexual dimorphism in this tooth. The observed sexual dimorphism in the permanent maxillary second molar may be related to its more distal position from the primary field of initiation and associated developmental influences. The findings suggest that shape variation in the maxillary second molar contributes to measurable differences between sexes within the studied population; however, further studies incorporating appropriate validation procedures are required before any conclusions regarding classification performance can be drawn.

Tooth shape, when analysed using standardized anatomical landmarks, allows precise and reproducible assessment of geometric features. Geometric morphometric analyses have previously been applied in orthodontic research to investigate occlusal relationships and dental morphology [33,35,36]. Although the present study was not designed to evaluate orthodontic outcomes, the observed patterns of shape variation may provide a basis for future investigations in this area. Tooth morphology is also widely used in evolutionary studies to understand variation and lineage patterns in human populations [37,38]. In addition, shape-based assessment using dental landmarks has been explored in forensic contexts for sex estimation, particularly when other methods are not feasible [39]. The present findings highlight that shape variation, particularly in the maxillary second molar, contributes to observable differences between sexes when analysed using landmark-based methods. However, the present study was not designed to evaluate forensic classification performance, and further validation studies would be required before any practical applications can be considered. Our results emphasize the importance of considering tooth morphology as a spatial and geometric construct rather than relying solely on linear measurements. The observed patterns provide insight into how specific regions of the tooth contribute to shape variation.

This study has several strengths. The use of three-dimensional geometric morphometric analysis allowed comprehensive evaluation of tooth shape independent of size, overcoming limitations of conventional linear odontometry. The inclusion of anatomically and geometrically defined landmarks provided a standardized and reproducible framework for shape analysis. Additionally, equal representation of males and females and the use of multiple multivariate statistical approaches enhanced the robustness and reliability of the findings.

The study has certain limitations. Although a modest age difference existed between male and female participants, its influence on the observed shape differences is likely to be minimal, as permanent molar crown morphology is established prior to the age range included in this study. The present study’s unicentric design and use of convenience sampling may limit the representativeness of the study population and reduce the generalizability of the findings to broader populations. Convenience sampling may introduce selection bias, as participants were recruited from a single clinical setting and may not fully reflect the variation present in the general population. Although efforts were made to include individuals from a defined geographic region, the presence of a mixed population with overlapping genetic pools could not be avoided. This was mitigated by including patients from the Dakshina Kannada region; nonetheless, for improved validity and precision, a multicentric study is required. The high classification accuracy observed in discriminant function analysis, despite non-significant group differences, may be influenced by the absence of cross-validation, and therefore could reflect potential model overfitting. As a result, the classification performance should be interpreted with caution. Further studies integrating genetic data with geometric morphometric analysis are required to better evaluate the relationship between genetic factors and variations in tooth morphology within populations.

In order to extract the ideal parameters from landmark data, future research in tooth shape analysis can investigate more advanced mathematical and computer models. In their 2020 study, Choi G et al. showed that quasi-conformational theory produced better results than area based and Procrustes based approaches for identifying sex and ancestry in Australian individuals with European heritage and those who are indigenous [40].

The findings of this study also provide a structured understanding of molar morphology through landmark-based analysis, which may aid in improving conceptual clarity of tooth shape variation and its determinants. Identifying specific regions that contribute to differences in shape can support a more systematic approach to teaching dental anatomy and morphology.

## Conclusion

The present study demonstrates that geometric morphometric analysis can identify sex-related differences in the shape of the maxillary molars, with significant variation observed in the second molar but not in the first molar. These findings indicate that geometric morphometric analysis can identify shape variation associated with sexual dimorphism in maxillary molars within the studied population. The developmental significance of these findings warrants further investigation, and future studies are needed to evaluate their potential relevance in orthodontic and forensic applications.
