# Supplementary material for: Geometric morphometric analysis of maxillary molar sexual dimorphism: A cross-sectional study
Source: PLoS One. 2026 Aug 7;21(8):e0355612. doi: 10.1371/journal.pone.0355612 (PMC13450795; doi:10.1371/journal.pone.0355612)
Supplement: Supplementary file 1 [file pone.0355612.s002.docx]

# Response to editor

| Editorial comment | Comment | Reply | Location in manuscript (without track changes) |
| --- | --- | --- | --- |
| Editorial | 1. Please ensure that your manuscript meets PLOS ONE's style requirements, including those for file naming. The PLOS ONE style templates can be found at  <https://journals.plos.org/plosone/s/file?id=wjVg/PLOSOne_formatting_sample_main_body.pdf> and  <https://journals.plos.org/plosone/s/file?id=ba62/PLOSOne_formatting_sample_title_authors_affiliations.pdf> | We thank the editor for this guidance. The manuscript has been revised to comply with PLOS ONE formatting requirements, including title page structure, headings, figure captions, and overall style. | Lines ~1–25  Title  Short title  Authors + affiliations |
|  | 2. Thank you for stating the following in the Acknowledgments Section of your manuscript:  “Study supported in part by a the Science and Engineering Research Board (SERB), Department of Science and Technology, Government of India (file number CRG/2020/001057)”  We note that you have provided funding information that is not currently declared in your Funding Statement. However, funding information should not appear in the Acknowledgments section or other areas of your manuscript. We will only publish funding information present in the Funding Statement section of the online submission form.  Please remove any funding-related text from the manuscript and let us know how you would like to update your Funding Statement. Currently, your Funding Statement reads as follows:  “Study supported in part by a the Science and Engineering Research Board (SERB), Department of Science and Technology, Government of India (file number CRG/2020/001057)”  Please include your amended statements within your cover letter; we will change the online submission form on your behalf. | We thank the editor for this clarification. The funding information has been removed from the manuscript file in accordance with journal requirements. The funding details have been provided in the cover letter and will be included in the Funding Statement through the submission system. |  |
|  | 3. We note that the grant information you provided in the ‘Funding Information’ and ‘Financial Disclosure’ sections do not match.  When you resubmit, please ensure that you provide the correct grant numbers for the awards you received for your study in the ‘Funding Information’ section. | We thank the editor for this observation. The grant information has been reviewed and corrected to ensure consistency across all submission fields. |  |
|  | 4. Thank you for stating the following financial disclosure:  “Study supported in part by a the Science and Engineering Research Board (SERB), Department of Science and Technology, Government of India (file number CRG/2020/001057)”  Please state what role the funders took in the study.  If the funders had no role, please state: "The funders had no role in study design, data collection and analysis, decision to publish, or preparation of the manuscript."  If this statement is not correct you must amend it as needed.  Please include this amended Role of Funder statement in your cover letter; we will change the online submission form on your behalf. | We thank the editor for this clarification. The role of the funder has been clearly stated in the cover letter in accordance with journal requirements. |  |
|  | 5. In the online submission form you indicate that your data is not available for proprietary reasons and have provided a contact point for accessing this data. Please note that your current contact point is a co-author on this manuscript. According to our Data Policy, the contact point must not be an author on the manuscript and must be an institutional contact, ideally not an individual. Please revise your data statement to a non-author institutional point of contact, such as a data access or ethics committee, and send this to us via return email. Please also include contact information for the third party organization, and please include the full citation of where the data can be found. | We thank the editor for this important clarification. The Data Availability Statement has been revised to comply with PLOS ONE policy. The dataset is now publicly available via Figshare, and the corresponding DOI has been provided in the manuscript. | Lines 445-448 |
|  | 6. Your ethics statement should only appear in the Methods section of your manuscript. If your ethics statement is written in any section besides the Methods, please delete it from any other section. | We thank the editor for this clarification. The ethics statement has been revised to appear exclusively within the Methods section, and any duplicate occurrences in other sections have been removed. | Lines 120-126 |
|  | 7. Please include a separate caption for each figure in your manuscript. | We thank the editor for this guidance. Separate and self-explanatory captions have been provided for all figures, formatted in accordance with PLOS ONE requirements. |  |
|  | 8. If the reviewer comments include a recommendation to cite specific previously published works, please review and evaluate these publications to determine whether they are relevant and should be cited. There is no requirement to cite these works unless the editor has indicated otherwise. | We thank the editor for this clarification. No specific references were suggested by the reviewers; however, the reference list has been reviewed and updated where appropriate to ensure relevance and consistency. |  |

# Response to reviewers

| Reviewer | Comment | Reply | Location in manuscript (without track changes) |
| --- | --- | --- | --- |
| Reviewer #1 | Title - Please add type of study. No capital letters, please consult some recently published Plos One papers.  "What do you mean when referring to "Shape as a Tool for Clinical Applications". I do not see any connections in the form of a "Tool for Clinical Applications". Please clarify. | We thank the reviewer for this suggestion. The title has been revised to include the study design and to conform to PLOS ONE formatting guidelines (sentence case). The revised title reads: ‘Geometric morphometric analysis of maxillary molar sexual dimorphism: a cross-sectional study’ | Line 4-5 title |
|  | Abstract - Please provide maximum information here. See Journal guidelines, the maximal word count is 300. Go to <https://journals.plos.org/plosone/s/submission-guidelines> , and revise carefully. | We have revised the abstract to improve completeness and clarity while remaining within the journal’s word limit. Additional methodological details (study design, landmarking approach, and statistical analyses) and key quantitative findings have been incorporated to enhance the informational value of the abstract. | Lines 31-56 |
|  | - You aimed to "investigate the geometric morphometric shape dimorphisms of the Permanent Maxillary First (UM1) and Second Molars (UM2)". Please stick to these aims, both with your results and with your conclusions. | We agree and have revised the abstract to ensure strict alignment between the stated aim (evaluation of sexual dimorphism in molar shape using geometric morphometry), highlighted at the end of the introduction, the results , and the conclusions (highlighted in grey). Statements not directly related to this aim have been removed. | Introduction line 115-118  Conclusion line 438-440 |
|  | - With regard to "(P<0.01)", please stick to Journal style. | All p-values have been reformatted according to journal style (e.g., p < 0.01, p = 0.150, p < 0.001). | Where ever applicable |
|  | - "Cognizance of the shape changes, asymmetry and allometry in molars is important for anthropological, developmental, clinical and forensic applications." Did you investigate "anthropological, developmental, clinical, or forensic applications"? I guess you did not. | We agree with the reviewer that these applications were not directly investigated in the present study. Accordingly, all such statements have been removed from the Abstract, Discussion, and Conclusion to avoid overinterpretation. |  |
|  | - With your Conclusions, please stick exclusively to your revised/adapted aims, and do not overstate. Do not simply repeat your results here. Do not speculate. Do not provide well-accepted (but unambiguous or even meaningless) phrases. Instead, provide a reasonable and generalizable extension of your outcome. | The conclusion has been revised to avoid repetition of results and speculative statements. It now provides a concise and generalizable interpretation strictly based on the findings of the study, focusing on the relevance of shape-based analysis for assessing sexual dimorphism. | line 438-440 |
|  | - This section has not been convincingly elaborated. | We have substantially revised the abstract for improved structure, clarity, and scientific precision, ensuring logical flow between objective, methods, results, and conclusion. | Lines 31-56 |
|  | Intro - "(...) but also enhance esthetics. [1]" Again, stick to Journal style, and consult some recently published Plos One papers. Citation style would be "(...) but also enhance esthetics [1]." Revise thoroughly. | We thank the reviewer for highlighting the citation formatting issues. All references have now been carefully revised to conform to PLOS ONE style, with citations placed before punctuation and without spacing errors. Redundant brackets and formatting inconsistencies have been corrected throughout the manuscript. | Throughout the manuscript |
|  | - "Interestingly, males and females have different patterns of distribution of Amelogenin, an enamel protein." With each and every fact, please provide sound references. | Reference 10 has been added for this statement. “Schwartz GT, Dean MC. Sexual dimorphism in modern human permanent teeth. Am J Phys Anthropol. 2005;128: 312–317. doi:10.1002/ajpa.20211” | Line 86 highlighted in blue |
|  | - "It is established that the X chromosome determines enamel thickness, whereas the Y chromosome influences both enamel and dentin thickness. [8]" What about females with XX chromosomes, having no Y chromosome? In these cases, dentin thickness will not be influenced? | We thank the reviewer for this important observation. We agree that the previous statement could imply that dentin thickness is dependent on the presence of the Y chromosome. This was not our intention. Dentin formation occurs in both males and females and is not exclusively influenced by the Y chromosome. The sentence has been revised to clarify that while the Y chromosome may contribute to variation in enamel and dentin dimensions, dentin development is not dependent on its presence. | Line 91-95 highlighted in green |
|  | - Neither aims nor objectives have been convincingly elaborated. Please provide a sound rationale. - "The objective of the present study is to explore the relationship between shape and size differences in tooth crowns, focusing on the hypothesis that males, due to the presence of both X and Y chromosomes, exhibit greater enamel and dentin thickness, thereby affecting the shape of the tooth crown more significantly." First, this is not a study proposal, please switch to past tense. Second, please provide a sound NULL HYPOTHESIS.- Same with "We hypothesize that molar shape, as assessed by landmark data in three dimensions, exhibits characteristic features of sexual dimorphism." Again, switch to past tense. Why do you repeat your information here? - Same with "The present study aims to determine the sexual dimorphism and discriminating capabilities of both shape and size of the maxillary permanent molars." Please revise carefully. | We thank the reviewer for this valuable suggestion. The aims and objectives have been revised to improve clarity and avoid repetition. The statements have been converted to past tense to reflect the completed nature of the study. Additionally, a formal null hypothesis has been included to strengthen the scientific framework of the study. | Highlighted in grey in the end of the introduction line 115 -118 |
|  | Meths - Please double check these sentence: "Abe K et al. (1996) reported the sexual dimorphism in the size of the human molars. [11] The upper first molars reported significantly larger sizes in males (first molar: 10.57±0.52; second molar: 9.98±0.58) compared to the females (first molar: 10.15±0.55; second molar: 9.47±0.60)." First, this section refers to methodology, so please do not mix with aspects sticking to a literature review. Second, what does "10.57±0.52" mean? Revise carefully. | We thank the reviewer for this suggestion. The sentence has been revised to clarify that the reported values represent mean ± standard deviation (in millimeters) of mesiodistal dimensions and were used solely for sample size estimation. The wording has been modified to maintain a methodological focus and avoid narrative interpretation | Line 145-151 highlighted in yellow |
|  | - Again, always stick to sound references, see "In 3D geometric morphometry, it is standard practice to ensure that the sample size is at least three times the number of variables being analyzed." | We thank the reviewer for this observation. We acknowledge that there is no fixed rule defining sample size in geometric morphometrics. The statement has been revised to reflect established principles that emphasize the need for sample size to exceed the number of variables for stable multivariate analysis. The reference to a fixed multiple has been removed, and the rationale has been clarified accordingly. | Line 157 to 159 Highlighted in yellow |
|  | - You sample size calculation would seem hard to follow. You have stated that "Based on the centroid values of males and females of the upper first premolar and upper second premolar", and it remains unclear why you have calculated with premolar values when going for a study on molars. | We thank the reviewer for this observation. The reference to premolar values in the sample size calculation was an oversight and has been corrected. The calculation was performed using previously reported data on mesiodistal dimensions of maxillary molars from Abe et al. (1996). The text has been revised to clearly reflect the use of molar data and to improve clarity and readability of the sample size estimation | Line 145 to 155 |
|  | - What about the pre-registration date and number of your study? | We thank the reviewer for this suggestion. As this was an observational cross-sectional study using existing clinical records, it was not pre-registered. This has now been clarified in the Methods section. | Line 125-126 highlighted in green |
|  | - With ALL materials and methodologies (including statistical software), please use general/non-proprietary names with your text, followed by (brand name; manufacturer, city, ST[ate - abbreviated, if US], country) in parentheses. Stick to semicolon. Please ensure reproducibility, and revise thoroughly. | We thank the reviewer for this suggestion. All materials and software have been revised to include generic names followed by brand name, manufacturer, city, and country in parentheses to ensure reproducibility in the materials and methods section | Line 164  Line 172  Line 196  Line 216 |
|  | - Aspects referring to your statistical analysis would seem missing. Remember that you have provided p values, and you have started with H0. | We thank the reviewer for this observation. A dedicated statistical analysis section has now been included. The statistical tests, level of significance, and null hypothesis have been clearly defined to improve clarity and reproducibility. | Line 216-231 |
|  | Results - "(...) but not in first molar (P value 0.1498). (Table 2)" Revise for sound full stops. | We have revised the same through out the manuscript |  |
|  | - "P" would seem clear and self-explanatory. Do not use "P value". - Please compare "(P value 0.1498)" and "(p =0.0012)", and revise for sound and uniform Journal style. Stick to 3-digit p values. | We thank the reviewer for this observation. All statistical reporting has been revised to ensure consistency with journal style. The term “P value” has been removed throughout the manuscript, and all p-values are now uniformly reported in lowercase as p, with appropriate spacing and rounded to three decimal places (e.g., p = 0.150, p = 0.001, p < 0.001 where applicable). |  |
|  | - Again, what does "The procrustes distances were 0.0229 and 0.0284" mean? | We thank the reviewer for this observation. Procrustes distance represents the magnitude of shape difference between group mean configurations after Procrustes superimposition. It is a unitless measure derived from landmark coordinates, where higher values indicate greater shape divergence between groups. The text has been revised in the results section to clarify this interpretation. | Line 256 Highlighted in yellow |
|  | - Another example would be "The distal marginal ridge is narrower in females (landmarks 11,27) and mesial marginal ridge was narrower in males (landmarks 5,21)." What does this mean? What is meant when referring to "narrower"? Do you refer to "significant differences" here? | We agree that the term “narrower” required clarification. In geometric morphometric analysis, such descriptions refer to relative positional differences of specific landmarks contributing to overall shape variation between groups. These observations are derived from discriminant function analysis and represent directional shape changes rather than direct linear measurements. The text has been revised to clarify that these differences reflect relative landmark displacement patterns between sexes and are interpreted in the context of overall shape variation | Line 275 to 304 |
|  | - Same with expressions like "more buccally placed", "more lingually placed", or "more cervically placed". - Please note that the aspects given above would not seem an exhaustive list. Revise carefully, but thoroughly. | We thank the reviewer for this important observation. The descriptive terms such as ‘narrower’, ‘more buccally placed’, and ‘more cervically placed’ have been revised throughout the manuscript to reflect their correct interpretation in geometric morphometric analysis. These terms now explicitly refer to relative displacement of landmarks and differences in spatial configuration rather than direct linear measurements. The text has been modified to clarify that these observations represent shape differences derived from discriminant function analysis and do not imply separate statistical testing for each individual feature | Line 275 to 304 |
|  | Disc - What about H0? | We thank the reviewer for this valuable suggestion. A statement addressing the null hypothesis has now been incorporated at the beginning of the Discussion section, clearly indicating its acceptance or rejection based on the study findings. | Line 306-310 |
|  | - "Butler’s field model (1939) and Osborn’s Clone model (1978) are the two theories that describe tooth formation." Reference numbers missing. | We thank the reviewer for pointing this out. The appropriate references have now been added to support this statement, and the sentence has been revised accordingly | Line 312 highlighted in blue |
|  | - Same with "A study by Batbayar et al. (2016), showed that in the absence of the maxillary first molar the second molar size is larger than the group of individuals with the presence of first molar." Please remember that "This manuscript is an updated submission of our work previously prepared for PLOS Computational Biology, now revised and formatted in accordance with the guidelines of PLOS ONE." Consequently, a sound piece of work would seem expectable, you surely will agree. | We thank the reviewer for this comment. The sentence has been revised for clarity, scientific tone, and precision, and the appropriate reference has been added. The wording has been improved to better reflect the underlying biological interpretation and to align with journal standards. | Line 320 highlighted in blue |
|  | - Same with "Morita et al. (2014) have shown that the hypocone has higher rates of variability as compared to (...)." Reference number missing. | We thank the reviewer for pointing this out. The appropriate reference has now been added to support this statement, and the sentence has been revised accordingly. | Line 330 unhighlighted reference [9] |
|  | - Same with "Developmental analysis showed that crown size is dependent on (...)" and with "If the duration of the cusp development takes longer time it does so at the expense of the later forming cusps." There are many mor references missing with your thoughts given with this section. | We thank the reviewer for this observation. The section has been revised to improve clarity of attribution. The statements derived from Morita et al. (2014) have been consolidated and appropriately referenced to avoid repetitive citation while ensuring that all interpretations are clearly supported by the same source. | Line 330-335 highlighted in grey |
|  | - "(...) there are studies, showing lack of sexual dimorphism of molars." Do you agree that the word studieS would call for referenceS? | We thank the reviewer for this observation. The sentence has been revised to avoid implying multiple studies where only a single reference is cited. The wording has also been refined for clarity and scientific accuracy. Also, the reference is cited | 361-363 highlighted in pink |
|  | - Again, revise for sound full stop and spacebar use, see "(...) of Romania. [22]The period of (...)". | Formatting revision has been done throughout the manuscript. |  |
|  | - What is the "mesiolingual cusp tips in maxillary second molar"? | We thank the reviewer for this observation. We agree that the phrase “cusp tips” was unclear and could lead to ambiguity. The sentence has been revised to remove the term “tips” and to clearly refer to the paracone (mesiobuccal cusp) and protocone (mesiolingual cusp) as anatomical regions of the crown. The revised sentence now more accurately conveys that differences in enamel thickness and developmental patterns between these cusps in the maxillary second molar, compared to the first molar, may contribute to the observed variation in sexual dimorphism. | 370 to 372 |
|  | - "The findings presented in this manuscript were also discussed at the 19th National Conference of The Indian Association of Forensic Odontology, conducted by G Pulla Reddy Dental College and Hospital, Kurnool, Andhra Pradesh." Would this be of any relevance here? | We thank the reviewer for this observation. The conference presentation has been cited (Reference 33) only where it directly contributes to the scientific discussion. Specifically, it supports the explanation of factors influencing cusp morphology, such as space availability, growth patterns, and surrounding tissue relationships, which are relevant to interpreting variation in molar shape. The reference has been used to strengthen the biological context of the findings rather than to report the presentation itself. | Lines 383-395 |
|  | - What about the strengths of your study? | We thank the reviewer for this suggestion. A dedicated section describing the strengths of the study has now been incorporated into the Discussion. | Lines 409-414 |
|  | - There would seem more limitations to be discussed. | We thank the reviewer for this observation. The limitations section has been expanded to provide a more comprehensive discussion of methodological and sampling constraints, along with directions for future research. | Lines 415-426 |
|  | - What about the responses to your teaching objectives? | We thank the reviewer for this comment. A paragraph has been added to highlight the interpretative and educational relevance of the findings, particularly in understanding tooth morphology through landmark-based analysis | Lines 432-436 |
|  | Concl - Again, with your Conclusions, please stick exclusively to your revised/adapted aims, and do not overstate. Do not simply repeat your results here. Do not speculate. Do not provide well-accepted (but unambiguous or even meaningless) phrases. Instead, provide a reasonable and generalizable extension of your outcome. - "In summary, the use of 3D shape coordinates provides a more comprehensive understanding of tooth morphology, with significant implications for dental restoration, orthodontics, and forensic identification." This has not been investigated, has it? | We thank the reviewer for this important observation. We agree that the statement extended beyond the scope of the present study and implied applications that were not directly investigated. This has been corrected. The conclusion has been revised to align strictly with the study aims and findings, and all references to broader clinical and forensic applications have been removed to avoid overstatement. | Lines 438-442 |
|  | Refs - Revise for sound and uniform Journal style. | We thank the reviewer for this observation. The reference list has been thoroughly revised to ensure consistency with the journal style. Formatting of journal names, punctuation, capitalization, and DOI presentation has been standardized throughout the manuscript. |  |
|  | Figs/Tables - Please revise for sound legends. The latter must be self-explanatory. | We thank the reviewer for this valuable suggestion. All figure and table legends have been revised to provide clearer, more detailed, and self-explanatory descriptions. The updated legends now include information on the methods, variables represented, and guidance for interpretation, ensuring that each figure can be understood independently of the main text. |  |
|  | In total, this revised and re-submitted draft would seem to suffer from numerous shortcomings. Please note that it would seem clear (and your surely will confirm) that all 6 Co-Authors have "read, reviewed, revised, and approved" this submitted draft. Consequently, a faultless draft would seem expectable. Moreover, some major drawbacks do not allow for proceeding with this submission. | We thank the reviewer for the candid and critical evaluation of our revised manuscript. We fully acknowledge that the manuscript required further refinement, and we have carefully addressed all the concerns raised. In this revision, we have undertaken a comprehensive review of the entire manuscript to improve methodological clarity, consistency in reporting, statistical transparency, and overall presentation.  All sections—including the Abstract, Methods, Results, Discussion, tables, and figures—have been thoroughly revised for accuracy, coherence, and language quality. Particular attention has been given to correcting inconsistencies, improving figure and table legends, standardizing statistical reporting, and addressing previously identified methodological concerns.  We confirm that all co-authors have reviewed and approved the revised version. We believe that the manuscript has been substantially strengthened through these revisions and now meets the expected standards for scientific rigor and clarity. |  |
| Reviewer #2: Review Comments to the Author | The manuscript explores an interesting and relevant topic, investigating sexual dimorphism in maxillary molars using 3D geometric morphometric analysis. The methodological approach is appropriate, and the use of landmark-based morphometry represents a strength of the study. However, several important issues need to be addressed to improve the scientific rigor, clarity, and overall impact of the manuscript. | We thank the reviewer for the positive evaluation of our work and for recognizing the relevance of the topic and the strengths of the methodological approach. We appreciate the constructive feedback provided to improve the scientific rigor, clarity, and overall impact of the manuscript. All the comments have been carefully considered, and the manuscript has been revised accordingly. Detailed responses to each of the specific points are provided below. |  |
|  | First, there is a noticeable mismatch between the title and the content of the study. While the title emphasizes clinical applicability, the manuscript provides limited discussion on how the findings can be translated into clinical practice. The authors are encouraged to expand the discussion by including concrete clinical scenarios (e.g., implications for orthodontic planning, restorative procedures, or occlusal reconstruction). Alternatively, the title should be revised to better reflect the actual scope of the study. | We thank the reviewer for this important observation. We agree that the earlier version of the title suggested broader clinical applicability that was not directly investigated in the present study. In line with this and to maintain consistency with the study aims and findings, the title has been revised to more accurately reflect the scope of the work. All references implying direct clinical or forensic applications have also been removed or moderated to avoid overstatement. | Lines 3-5 |
|  | Second, key sample characteristics are missing. The manuscript does not report the age distribution of the participants, which is a relevant variable in dental morphology due to potential effects of wear and age-related changes. This information should be included and discussed. | We thank the reviewer for this important observation. The age distribution of the study sample has now been included in the results section, with mean and standard deviation reported for both sexes. | Line 233-235 |
|  | Additionally, the study does not specify whether right or left molars were analyzed, nor whether bilateral symmetry was considered. | Both right and left maxillary molars were included in the analysis, and landmark coordinates from both sides were averaged to obtain a representative value for each individual, thereby minimizing the influence of side-related variation. | Lines 197-201 highighted in blue |
|  | Given that asymmetry is mentioned, this clarification is essential. | Although asymmetry is discussed conceptually in the context of dental morphology, it was not specifically analysed in this study, as the primary objective was to evaluate sexual dimorphism. This has now been clarified in the Methods section. | Line 200-201 |
|  | Third, the rationale for selecting only maxillary molars is not clearly justified. The authors should provide a scientific basis for this choice. | We thank the reviewer for this observation. The rationale for selecting maxillary molars has now been clarified in the Introduction. Maxillary molars were selected due to their distinctive occlusal morphology and well-documented variation in cusp and groove patterns, particularly the hypocone and cusp of Carabelli. In addition, the first and second maxillary molars exhibit comparable anatomical landmarks and functional characteristics, allowing for consistent landmark placement and reliable shape comparison, unlike mandibular molars, which show greater morphological variability. These features, along with their established use in morphometric studies, make them a suitable model for evaluating shape-based sexual dimorphism. | Lines 100-108 |
|  | Regarding methodology, although appropriate statistical approaches appear to have been used (e.g., Procrustes analysis, PCA, discriminant function analysis, and regression), the reporting is insufficient. Statistical tests such as ANOVA and regression should be clearly described in the Methods section, including assumptions, significance levels, and analytical procedures. | We thank the reviewer for this important observation. The statistical analysis section has been expanded to improve clarity, including specification of the analytical procedures and significance level.  A statement regarding standard assumptions of multivariate geometric morphometric analysis has also been incorporated to improve methodological transparency. | Line 219-220 |
|  | Additionally, no information is provided regarding intra- or inter-observer reliability for landmark placement, which is critical in geometric morphometric studies. | Additionally, a dedicated subsection on reliability assessment has now been included. Intra- and inter-observer reliability of landmark placement were evaluated using a random subsample, and intraclass correlation coefficients demonstrated excellent agreement. Measurement error related to digitization was also assessed using a permutation-based ANOVA approach and was found to be negligible. | Lines 204-215 Highlighted in yellow |
|  | The sample size calculation is presented; however, there are inconsistencies in the number of landmarks and variables considered (28, 18, and 16), which should be clarified | We thank the reviewer for this observation. The sample size section has been revised to improve clarity and remove statements suggesting a fixed rule. The description now reflects established multivariate principles, emphasizing that the sample size exceeds the number of variables to ensure stability of the analysis. | Lines 144-159 Highlighted in yellow in sample size section |
|  | . The use of convenience sampling should also be acknowledged as a limitation. | We have acknowledged the limitation in the third last paragraph of discussion as follows “The study has certain limitations. This study's unicentric design and convenience sampling may limit generalizability of findings to broader population.” | Line 415-416 Highlighted in green in limitation paragraph |
|  | Another important concern relates to the Discussion section. Several statements are presented without appropriate references, using expressions such as “many studies have shown” or “it is known that.” All scientific claims, particularly those related to genetic influence, tooth development, and sexual dimorphism, should be supported by relevant and preferably recent literature. | We thank the reviewer for this important observation. The Discussion section has been carefully revised to ensure that all scientific statements are supported by appropriate references. Generalized expressions have been removed or replaced with specific, evidence-based statements. In addition, two relevant references have been incorporated to strengthen the supporting literature where required. Sections that extended beyond the direct scope of the study have also been modified to avoid overinterpretation and to maintain alignment with the study aims. |  |
|  | Finally, the manuscript does not clearly ensure compliance with data availability requirements. The raw data underlying the analyses (e.g., landmark coordinates) are not explicitly provided or linked to a public repository. This should be addressed in accordance with journal policies. | We thank the reviewer for this observation. The underlying data, including landmark coordinate datasets, have now been made publicly available in a Figshare repository, and the Data Availability statement has been updated accordingly in compliance with journal requirements. | Lines 445-448 |
|  | In terms of language, the manuscript is generally understandable but would benefit from careful revision to improve clarity, grammar, and consistency. Some sentences are overly complex, and minor typographical and organizational issues are present. | We thank the reviewer for the constructive feedback. The manuscript has been carefully revised to improve clarity, grammar, and overall consistency. Sentences have been simplified where necessary, and typographical as well as organizational issues have been corrected. |  |
|  | In conclusion, the study has merit, but substantial revisions are required to improve methodological transparency, strengthen the discussion, and align the manuscript with its stated clinical relevance. | In response to the broader concerns, substantial revisions have been undertaken to enhance methodological transparency, including detailed reporting of sample characteristics, statistical procedures, and reliability assessment. The Discussion has been refined to ensure that all statements are appropriately supported and aligned with the study aims, and sections implying direct clinical applicability have been modified to avoid overstatement. We believe these revisions have significantly improved the clarity and scientific rigor of the manuscript. |  |
| Reviewer #3: | The manuscript addresses a relevant question and applies a potentially useful 3D geometric morphometric approach to maxillary molar sexual dimorphism. The study is of interest, but the manuscript requires substantial revision before it can be considered further. | We thank the reviewer for the positive evaluation of our work and for recognizing the relevance of the research question and methodological approach. We appreciate the constructive feedback provided to improve the manuscript. The manuscript has undergone substantial revision to address all the concerns raised, including improvements in statistical reporting, methodological transparency, and alignment between results and interpretation. Detailed responses to each specific comment are provided below. |  |
|  | First, the conclusions overstate the results. The manuscript concludes that both UM1 and UM2 can discriminate sex, but the reported analyses show a significant shape difference only for UM2, not UM1. This inconsistency appears in the abstract, results, and conclusion, and the claims should be revised to match the actual findings. | We thank the reviewer for this important observation. The manuscript has been revised to ensure consistency between the results and the conclusions. It is now clearly stated that statistically significant shape differences were observed only in the maxillary second molar, while the first molar did not show significant differences. The abstract and results sections have been updated accordingly to avoid overstatement, and the conclusions have been aligned with the actual findings. | Lines 438-440 highlighted in grey |
|  | Second, the discrimination analysis needs much clearer reporting. The manuscript reports about 95% classification accuracy, yet Table 4 shows non-significant P values, and the PCA is described as showing a homogeneous distribution of male and female samples. The authors should explain exactly how classification accuracy was obtained, and whether any cross-validation or independent validation was performed. Without that, the forensic and clinical claims are too strong. | We thank the reviewer for this important observation. The description of discriminant function analysis has been clarified in the Methods section. The reported classification accuracy was derived from the original dataset, and no cross-validation or independent validation was performed. This limitation has now been explicitly acknowledged.  In addition, the Results section has been revised to clarify that, despite high classification accuracy, the group differences were not statistically significant and principal component analysis showed overlap between sexes. The interpretation of classification results has been moderated accordingly to avoid overstatement.  Further lack of cross validation is also stated as a limitation of the study | Line 422 Highlighted in blue  Lines 260-263 (including table legend)  Line 422 highlighted in blue |
|  | Third, landmark reliability is not reported. Because the study depends on 28 manually placed 3D landmarks, intra-observer reproducibility, and ideally inter-observer agreement or digitization error, should be presented. This is important for judging the robustness of the morphometric findings. | We thank the reviewer for this important observation. A detailed reliability assessment has now been incorporated in the Methods section. Intra- and inter-observer reliability of landmark placement were evaluated using a random subsample, and intraclass correlation coefficients demonstrated excellent agreement (ICC = 0.982–1.000). In addition, digitization error was assessed using a permutation-based ANOVA approach and was found to be minimal relative to biological variation. These additions strengthen the methodological robustness of the study. | Lines 204-215 Highlighted in yellow |
|  | Fourth, the methods need clarification. The sample size justification is internally inconsistent and mixes different rationales. In addition, the study uses convenience sampling from a single center, which limits generalizability and should be more explicitly acknowledged in the interpretation. The manuscript should also clarify the unit of analysis, including how right and left molars were handled and whether bilateral observations were treated independently. | We thank the reviewer for this observation. The Methods section has been revised to improve clarity. The sample size calculation has been streamlined, and  the use of convenience sampling has been explicitly acknowledged as a limitation.  In addition, the handling of bilateral molars has been clarified. Both right and left molars were included, and averaged landmark coordinates were used to represent each participant. The unit of analysis has now been explicitly defined as the individual to avoid any ambiguity. | Lines 144-159 Highlighted in yellow  Lines 415-416 Highlighted in green under limitation section  Lines 197-203 Highlighted in blue and green |
|  | Fifth, the discussion and title should be more restrained. The title emphasizes “clinical applications,” and the discussion makes broad forensic and clinical inferences, but the present study is a single-center exploratory morphometric analysis. The practical implications should therefore be toned down unless supported more directly by the data. | We thank the reviewer for this important observation. The title has been revised to accurately reflect the scope of the study and no longer includes references to clinical or forensic applications. The Discussion section has also been carefully modified to avoid overstatement, and broader implications have been expressed in a more cautious and evidence-based manner, consistent with the exploratory nature of the study. |  |
|  | Finally, the manuscript would benefit from editorial revision for clarity and consistency. The use of “sex” and “gender” should be standardized, the keyword “innovation” is not informative, and the data availability statement should be made fully consistent with PLOS requirements, since the manuscript text states that data are available on request. | We thank the reviewer for this observation. The manuscript has been carefully revised to improve clarity, grammar, and overall consistency. Terminology has been standardized throughout, with the term “sex” used consistently in place of “gender.”  The keyword list has been refined by removing non-informative terms. In addition, the Data Availability statement has been updated to comply with journal requirements, with the underlying data now publicly available in a Figshare repository. | Throughout the manuscript |
|  | Overall, the study has potential, but the manuscript needs major revision in statistical reporting, methodological transparency, and interpretation before it is suitable for publication. | We thank the reviewer for the constructive and insightful feedback. The manuscript has undergone substantial revision to address all the concerns raised. Statistical reporting has been clarified, including detailed description of analytical procedures and appropriate interpretation of discriminant function analysis results. Methodological transparency has been improved through the addition of sample characteristics, clarification of the unit of analysis, and inclusion of a comprehensive reliability assessment.  The Discussion and conclusions have been carefully revised to ensure alignment with the study findings and to avoid overinterpretation. The title, abstract, and data availability statement have also been updated in accordance with journal guidelines. We believe that these revisions have significantly strengthened the clarity, rigor, and overall quality of the manuscript. |  |
| Reviewer #4: | The manuscript aims to evaluate the dimorphism of the first and second molars for clinical applications. It proposes an elaborate technique based on 3D x, y, and z landmark coordinates. However, numerous aspects require enhancement before being considered for publication. | We thank the reviewer for the overall assessment and constructive feedback. The manuscript has been extensively revised to address the concerns raised, as detailed in the responses below. |  |
|  | 1. The necessity of dimorphism for clinical application is unclear throughout the introduction and discussion. The introduction lacks a definitive application that would substantiate the objectives as a scientific need to be addressed by this research. What is the impetus for your inquiry? | We thank the reviewer for this insightful comment. The Introduction has been revised to better clarify the rationale of the study. The focus of the study is to provide a quantitative framework for evaluating shape-based sexual dimorphism using geometric morphometric methods, addressing limitations of conventional linear measurements. The emphasis has been refined to highlight the methodological and morphological significance of the work, rather than direct clinical application, in order to align with the scope of the study. | Lines 109-114 Highlighted in grey in introduction |
|  | 2. Moreover, I recognize that in forensic sciences, this would be pertinent; however, it is not referenced in the introduction, only cited at the conclusion of the debate. | We thank the reviewer for this observation. A brief statement has been incorporated in the Introduction to acknowledge the relevance of sexual dimorphism in dental morphology within forensic contexts. At the same time, the scope of the study has been clearly defined as focusing on shape analysis using geometric morphometric methods rather than direct forensic application, ensuring consistency throughout the manuscript. | Lines 115-118 Highlighted in grey |
|  | 3. Significant emphasis is placed on the embryological and genetic causes of diversity; nevertheless, minimal implementation of this knowledge occurs in practice. | We thank the reviewer for this valuable observation. The Discussion has been revised to improve balance between theoretical explanations and interpretation of the study findings. While relevant developmental and genetic perspectives have been retained to provide context, their emphasis has been moderated. Greater focus has been placed on interpreting the observed morphometric patterns and their significance within the scope of the study. | Lines 403-408 Discussion before strength in grey |
|  | 4. The introduction has extensive sections of text lacking citations. In many instances, three or four sentences conclude with a citation, although it remains unclear if all the preceding content belongs to a singular citation. The authors should provide citations for every assertions, even if they are reiterated. | We thank the reviewer for this important observation. The Introduction has been revised to ensure that each scientific statement is appropriately supported by relevant references. Sentences have been restructured where necessary to clearly associate citations with specific claims. In particular, sections discussing the influence of body size on tooth dimensions and the relative stability of tooth shape have been clarified and appropriately referenced to improve attribution and scientific rigor. | Lines 73-80 Highlighted in grey in introduction |
|  | 5. The objectives are lengthy and intertwined with the hypothesis explanation. This outlines the objectives and subsequently the aims of the research. It is imperative that the objectives remain prominent throughout the discussion and conclusions. | We thank the reviewer for this observation. The objectives and hypothesis have been revised to improve clarity and conciseness. The aim of the study and the null hypothesis are now presented separately and in a more streamlined manner to enhance readability and maintain focus throughout the manuscript. | Lines 113-118 Highlighted in grey in end of the introduction |
|  | 6. Kindly adhere to the Strobe statement sequence for the elucidation of methodology and outcomes. Commence with ethical considerations, followed by setting and sampling. The document begins with the sample size calculation, followed by an explanation of the requirements for participant recruitment. | We thank the reviewer for this suggestion. The Methods section has been reorganized in accordance with the STROBE guidelines. The sequence now begins with ethical considerations, followed by study setting, participant selection, sampling, sample size calculation, data acquisition, reliability assessment, and statistical analysis. This restructuring improves clarity and aligns the manuscript with standard reporting guidelines. | Reordering done in methods section |
|  | 7. The approaches must establish explicit inclusion criteria. Dental conditions, the absence of syndromes, and the analyzed population (specifically, why were only individuals with historical roots in this community selected, considering this may introduce a risk of bias?) | We thank the reviewer for this observation. The inclusion and exclusion criteria have been clarified in the Methods section. Individuals with craniofacial anomalies, syndromes, or systemic conditions affecting tooth development have now been explicitly excluded.  In addition, the rationale for selecting individuals from a defined geographic region has been clarified. This approach was intended to minimize population heterogeneity and reduce variability related to genetic and environmental factors. The potential limitation in terms of generalizability has also been acknowledged in the Discussion. | Lines 139-142 Highlighted in grey  Lines 137-139 Highlighted in yellow |
|  | 8. The age range serves as another illustration. It appears to involve minors, as shown by the acceptance of consent from legal guardians. Given that it is avoidable, why involve minor volunteers if the research could be conducted exclusively with legal age individuals? | We thank the reviewer for this important observation. The inclusion of younger participants was based on the presence of fully erupted permanent maxillary molars, as crown morphology is complete at this stage and not influenced by subsequent growth. The dental casts used in this study were obtained from routine orthodontic records, and no additional procedures were performed specifically for research purposes. All data were anonymized, and written informed consent for research use was obtained from participants or their legal guardians, as applicable. This clarification has been incorporated into the Methods section. | Lines 130-133 Highlighted in grey |
|  | 9. On page 7, line 160, substitute the phrase “convenience sampling upon their arrival” to eliminate ambiguity, as “convenience sampling” typically refers to a participant selection based on convenience or availability rather than calculation, as employed in this research. | We thank the reviewer for this observation. The description of sampling has been revised to improve clarity. The manuscript now specifies that participants were selected using convenience sampling from patients meeting predefined inclusion criteria during the study period, thereby avoiding ambiguity in terminology. | Line 142-143 Highlighted in green |
|  | 10. A critical aspect of the methodologies is the explicit delineation of the training process to guarantee reproducibility. The analysis must encompass both intra- and interobserver assessments to corroborate the findings, given that the study addresses a limited range of variance (sometimes within a 1mm threshold). | We thank the reviewer for this important observation. A detailed reliability assessment has been included in the Methods section, demonstrating both intra- and inter-observer agreement with excellent reliability (ICC = 0.982–1.000). In addition, digitization error was assessed and found to be minimal.  To further improve clarity, a statement regarding observer training and calibration prior to landmark placement has now been incorporated, ensuring reproducibility of the landmarking process. | Lines 205-215 Highlighted in yellow  Line 204-205 Highlighted in Grey |
|  | 11. A more comprehensive description of the statistical analysis should be provided in the techniques section. | We thank the reviewer for this observation. The statistical analysis section has been expanded to provide clearer descriptions of the analytical procedures used, including Procrustes superimposition, principal components analysis, Procrustes ANOVA, regression analysis, and discriminant function analysis. The purpose of each method and the interpretation of results have been clarified, and the significance level has been explicitly stated. In addition, the limitations of classification analysis in the absence of cross-validation have been acknowledged. | Lines 216-231 |
|  | 12. The results lack descriptive statistics, limiting comparisons of tooth size and shape with existing or future investigations. The verification of dispersion normalcy must be conducted for each measurement set. | We thank the reviewer for this observation. The primary outcome of the present study was shape variation analysed using geometric morphometric methods. As Procrustes shape coordinates represent multivariate data, conventional descriptive statistics are not directly applicable. Accordingly, shape variation was assessed using Procrustes-based methods, including principal components analysis and Procrustes ANOVA. |  |
|  | 13. It also failed to present some results, including the R², Spearman correlation, and other analyses. A confusion matrix can convert the results into percentages of specificity and sensitivity of the method. Additionally, AUC, collinearity analysis, VIFF, and other statistical components of logistic regression could enrich the results. | We thank the reviewer for these suggestions. The present study is based on geometric morphometric analysis, where shape is represented by multivariate Procrustes coordinates. Accordingly, statistical analysis was performed using methods appropriate for shape data, including Procrustes ANOVA, principal components analysis, regression for allometry, and discriminant function analysis.  Measures such as R², Spearman correlation, variance inflation factor (VIF), and logistic regression-based diagnostics (including AUC and confusion matrices) are typically applied in univariate or regression-based classification models and are not directly applicable to Procrustes-based shape analysis.  Discriminant function analysis was used to assess classification based on shape variables, and its interpretation has been clarified and moderated in the revised manuscript. |  |
|  | 14. In-depth application analysis. If a regression analysis was conducted, why not provide an equation or methodology for sex classification based on your findings? | We thank the reviewer for this observation. In the present study, regression analysis was performed to evaluate allometric relationships between size and shape, rather than for predictive classification. Sex classification was assessed using discriminant function analysis based on multivariate shape variables.  As geometric morphometric data involve high-dimensional shape coordinates, classification is not represented by a single regression equation but rather by multivariate discriminant functions. This distinction has been clarified in the Methods section to avoid ambiguity. | Lines 223-225 Highlighted in blue under statistics |
|  | 15. The discussion excessively focuses on the genetic factors and embryological origins of diversity, rather than addressing the practical implications of this discovery. | We thank the reviewer for this valuable observation. The Discussion has been revised to achieve a better balance between theoretical explanations and interpretation of the study findings. While relevant developmental and genetic perspectives have been retained to provide context, their emphasis has been moderated. Greater focus has been placed on the interpretation of morphometric findings and their relevance within the scope of the study, avoiding overstatement of direct applications. |  |
|  | 16. Minimal comparisons are made with other studies, and numerous references in the debate predate the threshold of 2020. | We thank the reviewer for this observation. The reference list has been updated to include recent studies where available. However, it is important to note that research specifically addressing sexual dimorphism in maxillary molars, particularly using geometric morphometric approaches, remains limited. As a result, several foundational references predate 2020 and have been retained due to their continued relevance in explaining tooth development, morphometric methodology, and sexual dimorphism.  Where appropriate, recent studies have been incorporated to complement these foundational works and provide contemporary context. |  |
| Reviewer #5: | The geomorphometric dental studies presented in this article have a broad applicability. Methods that allow identification of sex or account for sex-related differences in tooth development can be useful for odontologists, forensic scientists, and bioarchaeologists when it is not possible to determine biological sex using conventional methods. | We thank the reviewer for the positive appraisal of our work and for highlighting the broader relevance of geometric morphometric approaches in dental research. We appreciate the recognition of the potential applicability of such methods in fields such as odontological research, forensic science, and bioarchaeology. |  |
|  | The submitted article meets the requirements for a scientific publication. It is written in fluent language; the problem is clearly stated; appropriate statistical methods are used; the results are presented clearly; and the tables and figures are informative. The discussion is thorough, and the conclusions are consistent with the results. | We thank the reviewer for the encouraging and positive evaluation of our manuscript. We appreciate the acknowledgment of the clarity of presentation, appropriateness of the methodology, and consistency between the results and conclusions. |  |
|  | However, the topic of the article is sexual dimorphism in the size and shape of molars, which is also stated as the aim of the study. Nevertheless, this main topic is not reflected in the title. Although sexual dimorphism is mentioned in the short title, emphasizing it in the main title would help readers better grasp the article's main issue. | We thank the reviewer for this valuable suggestion. The title has been revised to explicitly reflect the primary focus of the study on sexual dimorphism. This change improves clarity and ensures that the title accurately represents the aims and findings of the manuscript. | Lines 3-5 Title is changed |
|  | As for the sample size calculation, the determination is based on the publication by Abe K et al. (1966). Why was this particular study selected? | We thank the reviewer for this observation. The study by Abe K et al. was selected for sample size estimation as it provides quantitative data on sexual dimorphism in the mesiodistal dimensions of maxillary molars, which closely aligns with the tooth type and variables investigated in the present study. Despite being an earlier study, it remains a relevant and frequently cited reference in dental morphometric research.  Given the limited availability of studies reporting comparable quantitative data specifically for maxillary molars, this study was considered appropriate for estimating sample size. This clarification has been incorporated into the Methods section. | Lines 145-159 |
|  | The following remarks are more of an editorial nature:  •In general, the revision of the article by a native English speaker is recommended. | We thank the reviewer for this suggestion. The manuscript has undergone careful language editing to improve clarity, grammar, and overall readability. |  |
|  | •In cases when a reference or table number is cited at the end of a sentence, the dot is placed before the parentheses. Below are a few examples: Line 87: “These changes are most easily observed in human molars. [2]” Line 174: “…using slicermorph software (https://github.com/SlicerMorph, Seattle, USA). [15] (Table 1, Figure 1,2)” This style of formatting makes the text difficult to read and should be changed to a more common style where the period is placed after the parentheses, unless such a style is specified in the journal’s requirements. | We have revised the manuscript throughout so that the full stop is placed after the references in parenthesis. |  |
|  | •The use of articles when referring to “the first molar” and “the second molar” needs to be checked. Such definite articles are missing not once in the article. | We thank the reviewer for this careful observation. The manuscript has been thoroughly revised to ensure consistent and appropriate use of definite articles when referring to specific teeth, particularly “the first molar” and “the second molar.” All relevant sections, including the Abstract, Results, Discussion, and Conclusion, have been carefully checked and corrected to improve grammatical accuracy and clarity. |  |
|  | •When presenting the list of landmarks in the Results section, it is recommended to separate the numbers with spaces: e.g., change “landmarks 11,27” to “landmarks 11, 27” (line 2016). | We thank the reviewer for this helpful suggestion. The manuscript has been revised to ensure consistent formatting of landmark numbers, with appropriate spacing applied throughout the Results section and wherever applicable. |  |
|  | •In two paragraphs (lines 113–122 and 175–176), the text is written in a smaller font than in the rest of the article. | We thank the reviewer for this observation. The formatting inconsistency has been corrected, and the font size has been standardized throughout the manuscript to ensure uniform presentation. |  |
|  | •The p-values in the article are presented in three different formats: “P value 0.01” (lines 169, 246, 247), “p=0.0012” (line 200), and “p<0.001” (line 201). The presentation of the p-values needs to be standardized. | We thank the reviewer for this important observation. The presentation of p-values has been standardized throughout the manuscript to ensure consistency in reporting. As a result of revisions, the line numbers have changed accordingly. |  |
|  | •Line 133: change to “…the required sample size in each group...”  •Line 142: change to “…resulting in a total of 120 individuals.” | The changes have been done and highlighted in blue | Lines 155-57 Highighted in blue |
|  | •Lines 196 and 348: the term “gender” refers to a social construct, and should be changed to the biological term “sex”. | We thank the reviewer for this important clarification. The terminology has been revised throughout the manuscript, and all instances of “gender” have been replaced with the appropriate biological terms “sex” or “sexes,” ensuring consistency and accuracy. |  |
|  | •Line 205: change to “…discriminating between the sexes...” | We thank the reviewer for this suggestion. The sentence has been revised accordingly to improve clarity and consistency in terminology. |  |
|  | •Line 217: change to “..noted was that the occlusal table…” | The word “that” was included in the sentence as suggested. | Line 282 |
|  | •Line 234: the mentioned authors (Butler’s field model (1939) and Osborn’s Clone model (1978)) are not mentioned in the References. | The reference 11, 22 and 23 have been included as a reference for the same. | Line 312 |
|  | •Line 242: change to “…size is larger than in the group...” | The paragraph is completely altered as per the suggestions of another reviewer. |  |
|  | •Lines 248-249: clarify the sentence “The significant difference in the second molar is predominantly attributed due to extent of development the hypocone (distopalatal cusp) development.” | We thank the reviewer for this observation. The sentence has been revised to improve clarity and precision. The updated text now clearly states that the observed shape difference in the second molar is predominantly related to the extent of hypocone (distopalatal cusp) development, and further explains that the distopalatal region exhibits the greatest variation in shape. This has been supported with appropriate reference to Morita et al. (2014). | Line 325-330 |
|  | •Line 262: change to “...systems like functional, developmental, and/or genetic factors…” | The sentence has been modified accordingly. | Line 341 Highlighted in blue |
|  | •Line 284: change to “…to our results for the first molar.” | We have replaced the “of” to “for” as suggested. | Line 363 in pink |
|  | •Line 309: change to “…multiple factors that influence the shape…” | The word “that” has been incorporated to improve the grammar of the sentence. | Line 388 Highlighted in blue |
|  | •Line 316: change to “...to discriminate between sexes in the modern human.” | The word “between” has been added to improve the meaning of the sentence. | Line 395 Highlighted in blue |
|  | •Line 343: clarify the sentence “To further understand the genetic basis of tooth morphogenesis within the population, more study is required…” | We thank the reviewer for this observation. The sentence has been revised to improve clarity and specificity. It now explicitly states that future studies integrating genetic data with morphometric analysis are required to better understand the relationship between genetic factors and tooth morphology. | Lines 424-426 Highlighted in green |
|  | •Line 359: change to “...from the institutional ethics…” | The sentence has been formatted in sentence case to remove the first letter capitalization. | Line 124 Highlighted in blue |
|  | •Correct the use of capital letters and articles in Tables. For example: “Landmarks based on cusp” but “Landmarks based on Ridges” or “Buccal crest of curvature on mesiobuccal lobe”, but “Buccal crest of curvature on the Distobuccal lobe” (Table 1) “Maxillary First molar” (Table 2), but “Maxillary First Molar” (Table 3) “Prediction accuracy Female” but “Prediction Accuracy Male” (Table 4) | We thank the reviewer for this observation. All tables have been carefully revised to ensure consistency in the use of capitalization and articles. A uniform sentence-case format has been applied throughout, and terminology has been standardized to maintain clarity and consistency across all tables. | Lines 184-187 (Table 1)  Line 243-244 (Table 2)  Line 250-252 (Table 3)  Line 262-264 (Table 4) |
|  | •Table 4: some statistical values in the second column are in bold. Is that necessary? | We thank the reviewer for this observation. The formatting has been revised, and the bold formatting of statistical values in Table 4 has been removed to ensure uniform and consistent presentation. | Line 262-264 (Table 4) |
|  | In summary, the comments provided do not diminish the scientific value of the article. Once the article has been revised, it is definitely suitable for publication. | We sincerely thank the reviewer for the positive and encouraging assessment of our work. We appreciate the constructive feedback provided, which has helped improve the clarity and presentation of the manuscript. |  |
| Reviewer #6 | While the topic is relevant, the manuscript contains fundamental methodological and statistical flaws that invalidate the central findings. These are not issues amenable to revision; they would require the study to be substantially redesigned. The key reasons for rejection are outlined below. | We thank the reviewer for the detailed and critical evaluation of our manuscript. We respectfully clarify that the concerns raised primarily relate to aspects of reporting, interpretation, and clarity rather than fundamental flaws in study design. The study employs established geometric morphometric methods, including Procrustes superimposition, principal components analysis, and Procrustes ANOVA, which are appropriate for analysing shape variation.  In response to the reviewer’s concerns, the manuscript has been substantially revised to improve methodological transparency, clarify statistical procedures, and appropriately moderate the interpretation of findings. Specific issues related to classification analysis, sample size justification, measurement reliability, and data handling have been addressed in detail in the revised manuscript and corresponding responses below. |  |
|  | The claimed 95% sex classification accuracy is almost certainly an artefact of overfitting. With 84 shape variables and only 120 specimens (60 per sex), the variable-to-sample ratio (~1:1.5) is far below accepted thresholds (1:5–1:10). | We thank the reviewer for this important observation. We agree that the high classification accuracy observed in discriminant function analysis should be interpreted with caution, particularly in the absence of cross-validation and given the dimensionality of the shape data relative to the sample size.  In the revised manuscript, this has been explicitly acknowledged as a limitation, and the interpretation of classification performance has been appropriately moderated. The discriminant function analysis was used primarily as an exploratory tool to assess patterns of separation between groups based on shape variables, rather than as a predictive model for clinical or forensic application.  Accordingly, claims regarding classification accuracy have been revised to avoid overinterpretation, and the potential influence of overfitting has been clearly stated in the Discussion. | Line 227, Line 263 and Line 422  Line 422 |
|  | The abstract reports UM2 shape as “P<0.01” while Table 2 shows P=0.01 exactly. | We thank the reviewer for this observation. The reporting of p-values has been standardized throughout the manuscript to ensure consistency, and the values have been corrected to accurately reflect the statistical results. | Lines 47, 240 and 324 |
|  | The inconsistent use of “sex” versus “gender” further reflects a lack of rigour in reporting. | In addition, the terminology has been revised, and all instances of “gender” have been replaced with the appropriate biological term “sex” or “sexes” throughout the manuscript. |  |
|  | No intra- or inter-observer error analysis is reported for the 28 landmarks (10 of which are geometrically, not anatomically, defined). Without measurement error quantification, it is impossible to determine whether reported shape differences reflect biology or digitisation noise. This is a basic requirement in geometric morphometric studies. | We thank the reviewer for this important observation. In response, a detailed assessment of measurement reliability has now been incorporated into the Methods section in the revised manuscript. Intra- and inter-observer agreement for landmark placement were evaluated using a random subsample of 20 dental models. The intraclass correlation coefficient (ICC) ranged from 0.982 to 1.000, indicating excellent reliability.  In addition, digitization error was assessed using a permutation-based approach, and the variance attributable to measurement error was found to be less than 10%, indicating that it was negligible relative to biological variation. This addition strengthens the methodological robustness of the study. | Lines 204-215 Highlighted in yellow in the second last paragraph of the methods section |
|  | Sample size calculation is based on premolar centroid size data, not molar shape data, making it inappropriate for the study’s primary analyses. Convenience sampling from orthodontic patients introduces selection bias, as these patients may not represent normal dental morphology. | We thank the reviewer for this observation. We acknowledge that there was an oversight in the earlier version of the manuscript regarding the description of the sample size calculation. This has now been corrected and clearly rewritten in the revised manuscript to reflect that the estimation was based on mesiodistal dimensions of maxillary molars, which are relevant to the teeth studied.  Given the limited availability of prior studies reporting quantitative shape-based parameters for molars, size-based data were used as a proxy for sample size estimation. This approach has now been clarified in the Methods section.  In addition, the issues related to convenience sampling and bilateral data handling have been explicitly addressed and clarified in the revised manuscript to improve methodological transparency. | Lines 145-159 |
|  | The manuscript does not specify which side (left/right) was used or how bilateral data was handled, raising concerns about data independence. | Regarding bilateral data, this has now been clearly described in the Methods section. Both right and left maxillary molars were included, and landmark coordinates were averaged for each individual to minimize side-related variation. The unit of analysis was therefore the individual, and bilateral asymmetry was not analysed separately, as the primary objective was to evaluate sexual dimorphism.  These clarifications have been incorporated to improve methodological transparency. | Line 197-203 |
|  | The Discussion is disproportionately long and speculative. Figures are difficult to interpret and lack proper axis labels. The data availability statement contradicts PLOS ONE’s open-data policy. Multiple grammatical errors persist throughout. | We thank the reviewer for these constructive suggestions.  The Discussion section has been revised to improve focus and reduce speculative statements, with greater emphasis placed on interpretation of the study findings within the scope of the data.  Figures have been reviewed and updated to improve clarity and interpretability, including refinement of labels and descriptions where necessary.  The data availability statement has been corrected in accordance with PLOS ONE requirements, and the complete dataset has been made publicly accessible via the Figshare repository.  In addition, the manuscript has undergone careful language revision to improve grammatical accuracy, clarity, and consistency throughout. |  |
