# Supplementary material for: Geometric morphometric analysis of maxillary molar sexual dimorphism: A cross-sectional study
Source: PLoS One. 2026 Aug 7;21(8):e0355612. doi: 10.1371/journal.pone.0355612 (PMC13450795; doi:10.1371/journal.pone.0355612)
Supplement: Supplementary file 2 [file pone.0355612.s003.docx]

# Response to editor and reviewers

| Editorial comment | Comment | Reply | Location in manuscript (without track changes) |
| --- | --- | --- | --- |
| Editorial | A letter that responds to each point raised by the academic editor and reviewer(s). You should upload this letter as a separate file labeled 'Response to Reviewers'. | We thank the Academic Editor and reviewers for their valuable comments. A detailed point-by-point response addressing all comments and suggestions has been prepared and uploaded as a separate file entitled "Response to Reviewers". |  |
|  | A marked-up copy of your manuscript that highlights changes made to the original version. You should upload this as a separate file labeled 'Revised Manuscript with Track Changes'. | A marked-up version of the revised manuscript showing all modifications using track changes has been prepared and uploaded as a separate file entitled "Revised Manuscript with Track Changes". |  |
|  | An unmarked version of your revised paper without tracked changes. You should upload this as a separate file labeled 'Manuscript'. | A clean revised version of the manuscript with all changes accepted and without track changes has been prepared and uploaded as a separate file entitled "Manuscript". |  |

# Reviewer 1

| Editorial comment | Comment | Reply | Location in manuscript (without track changes) |
| --- | --- | --- | --- |
| Reviewer #1 | The previous Reviewers' comments have considerably improved this revised and re-submitted draft. All comments have been addressed, and all recommendations have been followed. In total, the Co-Authors responses would seem satisfying, and this manuscript is considered ready to proceed. | The authors sincerely thank the reviewer for the positive evaluation of the revised manuscript. We appreciate the reviewer’s recognition that the previous comments and recommendations have been satisfactorily addressed. We are grateful for the time and effort invested in reviewing our work and for the constructive feedback that helped improve the quality and clarity of the manuscript. |  |

# Reviewer 2

| Editorial comment | Comment | Reply | Location in manuscript (without track changes) |
| --- | --- | --- | --- |
| Reviewer #2 | The manuscript shows significant improvement compared to the previous version, particularly regarding the alignment between title, objective, methodology, results, and conclusion. The focus on shape analysis using geometric morphometric analysis has become more consistent throughout the manuscript, and there has been an important improvement in the cautious interpretation of the findings, especially after inclusion of the limitations related to the absence of cross-validation and the overlap observed in the PCA. Nevertheless, some methodological and interpretative weaknesses still remain, particularly regarding discriminant function analysis (DFA), potential overfitting and lack of validation, biological inferences extending beyond the data actually produced, the excessively narrative character of the discussion, and a conclusion that remains slightly assertive in some aspects. | The authors sincerely thank the reviewer for the careful evaluation of the revised manuscript and for recognizing the substantial improvements made in response to the previous review comments. We appreciate the positive assessment regarding the improved alignment between the study objectives, methodology, results, and conclusions, as well as the more consistent focus on geometric morphometric shape analysis.  We also acknowledge the reviewer’s remaining concerns regarding the interpretation of discriminant function analysis, the possibility of overfitting due to the absence of cross-validation, certain biological interpretations that may extend beyond the direct evidence generated by the study, the narrative nature of parts of the discussion, and aspects of the conclusion. These points have been carefully considered and addressed in the revised manuscript. Specific modifications have been made to further clarify the limitations of DFA, temper biological inferences where appropriate, improve the focus and structure of the discussion, and refine the conclusions to ensure they remain fully supported by the data presented. Detailed responses to each concern are provided below. |  |
| Introduction: | Introduction: - Some sections remain excessively extensive, particularly those related to genetic, chromosomal, and dental developmental mechanisms, which were not directly evaluated in the study; | The authors thank the reviewer for these constructive suggestions. We agree that certain portions of the Introduction could be further streamlined to improve focus and readability.  Accordingly, the sections discussing genetic, chromosomal, and developmental mechanisms have been condensed and revised to provide only the background necessary to contextualize the study while avoiding detailed mechanistic interpretations that were not directly investigated. | Line 83 to 89 |
| Introduction: | - The first paragraph still appears overly focused on digital scanning technologies and prosthetic/aesthetic applications, topics that do not directly represent the primary focus of the research; | The opening paragraph has also been modified to reduce emphasis on digital scanning technologies and prosthetic applications and to more directly introduce the rationale for geometric morphometric evaluation of molar shape. | Line 62-65 |
| **Introduction:** | - There is slight redundancy in sections discussing “shape versus size”; | In addition, the discussion of shape versus size has been reviewed and edited to eliminate redundancy and improve narrative flow. | Line 72-77 |
| **Introduction:** | - Consolidating references at the end of conceptual blocks could improve readability and avoid excessive citation repetition; | We agree that consolidating references at the end of conceptual blocks may improve readability. However, during the previous round of review, another reviewer expressed concern that several statements within the Introduction lacked explicit citation support and recommended that references be provided for individual assertions rather than only at the end of longer paragraphs. In response to that recommendation, citations were intentionally repeated throughout the text to ensure that each statement was clearly supported by the relevant source and to avoid ambiguity regarding the attribution of preceding content.  To balance readability and citation transparency, we have carefully reviewed the Introduction and consolidated citations where appropriate without compromising the traceability of supporting references. If the Editor or reviewers prefer a more extensively consolidated citation style, we would be pleased to make further modifications accordingly. | Introduction segment |
| **Methodology:** | Methodology: - The expression “obtained prospectively from orthodontic patient records” may create ambiguity between prospective data collection and retrospective analysis of existing clinical records. Clarifying the temporal process of data acquisition could improve methodological transparency; | We agree that the original wording could create ambiguity regarding the temporal nature of data collection. The sentence has been revised to clarify that the pretreatment dental casts were routinely collected as part of orthodontic records during the study period and were subsequently included in the present investigation. This revision improves methodological transparency and more accurately reflects the process of data acquisition. | Line 128-129 |
| **Methodology:** | - The justification for population homogeneity based on individuals from the same region could be presented more objectively and scientifically; | We agree that the rationale for selecting individuals from the same geographic region should be presented more objectively. The manuscript has been revised to clarify that the purpose of this criterion was not to assume population homogeneity, but rather to reduce potential variability arising from geographic and population-level differences. The revised wording emphasizes that this approach was intended to improve consistency within the study sample while acknowledging that complete genetic or population homogeneity cannot be guaranteed. | Line 133-138 |
| **Methodology:** | - Convenience sampling was appropriately declared; however, its potential biases could be acknowledged more explicitly; | We agree that convenience sampling may introduce selection bias and may limit the representativeness of the study population. Although convenience sampling was reported in the Methods section, the manuscript has been revised to more explicitly acknowledge its potential influence on external validity and generalizability. This limitation has now been emphasized in the Discussion and Limitations section. | Line 437-439 |
| **Methodology:** | - Sample size calculation was based on linear odontometric measurements, whereas the primary outcome of the study involves multivariate geometric shape analysis. Better methodological alignment or statistical justification could strengthen this section; | The authors thank the reviewer for this important observation. We agree that the sample size calculation was based on previously reported linear odontometric measurements rather than multivariate geometric morphometric shape variables. As no established sample size estimation approach was available for the specific three-dimensional landmark-based geometric morphometric analyses employed in this study, linear odontometric data were used as a pragmatic reference to guide sample size determination. The manuscript has been revised to clarify this point and to avoid implying that the calculation represents a formal power analysis for geometric morphometric inference. The final sample size is therefore presented as a practical approximation that supported the exploratory multivariate analyses undertaken in the study. | Line 155-161 |
| **Methodology:** | - Information regarding landmark reproducibility remains absent, including intra/interexaminer error assessment, calibration, and landmark reliability evaluation; | In the revised manuscript, a dedicated section describing observer calibration, intra- and inter-observer reliability assessment, and digitization-error analysis has been included in the Methods section.  Briefly, all observers underwent calibration and training prior to data collection. Reliability was assessed using a random subsample of 20 dental models. Inter-observer reliability was evaluated through independent landmark digitization by two observers, while intra-observer reliability was assessed through repeated landmark placement by a single observer after a five-day interval. Reliability was quantified using global intraclass correlation coefficients (ICC), which ranged from 0.982 to 1.000, indicating excellent agreement. In addition, digitization error was evaluated using repeated landmark digitization and a one-way nonparametric ANOVA with a randomized permutation procedure in MorphoJ software, demonstrating that measurement error accounted for less than 10% of total variance.  To further improve clarity, the description of these procedures has been expanded in the revised Methods section. | Line 209-224 |
| **Methodology:** | - There is some redundancy between the ethics and consent sections. | We agree that certain details relating to ethical approval and informed consent were repeated within the methodology section. The text has been revised to reduce redundancy by consolidating information regarding ethical approval, informed consent, and use of clinical records while retaining all information necessary for transparency and compliance with ethical reporting standards. | Line 122-127 |
| **Results:** | Results: - Interpretation of the high discriminant accuracy (~95%) still requires caution, particularly considering: lack of statistical significance in part of the results; - overlap between groups in the PCA; - absence of cross-validation; - The absence of cross-validation represents an important methodological limitation and likely contributes to overestimation of classification accuracy; | The authors thank the reviewer for this important observation and agree that the classification results should be interpreted cautiously. Although discriminant function analysis produced high apparent classification accuracy, the analysis was performed on the original dataset without cross-validation, and therefore does not represent validated predictive performance. Furthermore, principal component analysis demonstrated substantial overlap between male and female groups, and statistically significant shape differences were observed only for the maxillary second molar.  To address this concern, the manuscript has been revised to substantially reduce emphasis on classification accuracy. The Abstract, Results and Discussion sections have been modified to clarify that the discriminant function analysis was exploratory in nature and that the reported classification performance should not be interpreted as evidence of generalizable sex-classification capability. Additional statements highlighting the lack of cross-validation and the overlap observed in PCA have been incorporated where appropriate. | Line 48-50 (abstract)  Line 236-237 & line 270-272 (results)  Line 443-445 (discussion) |
| **Results:** | - Some anatomical interpretations related to landmark displacement remain excessively descriptive and could be synthesized to improve objectivity; | We agree that certain portions of the anatomical description associated with landmark displacement were overly detailed. The Results section has been revised to provide a more concise and objective summary of the principal shape differences observed between groups, while preserving the key findings derived from the geometric morphometric analysis. Detailed landmark-by-landmark descriptions have been condensed to improve readability and maintain focus on the major patterns of shape variation. | Lines 286-315 |
| **Results:** | - The results section could be slightly condensed, particularly regarding detailed anatomical descriptions of landmarks. | We agree that a more condensed presentation of landmark displacement patterns could improve readability. However, during a previous round of peer review, a reviewer specifically requested a more detailed anatomical description of the landmark configurations contributing to the observed shape differences. In response to that recommendation, the Results section was expanded to provide a clearer description of the anatomical regions associated with landmark displacement and to facilitate interpretation of the geometric morphometric findings.  To balance detail with readability, we have reviewed the text and ensured that the descriptions remain focused on the principal patterns of shape variation rather than speculative interpretation. At present, we have retained the landmark descriptions to address the concerns raised during the earlier review process. Nevertheless, should the Editor or reviewers prefer a more concise presentation, we would be pleased to further condense these sections. | Lines 286-315 |
| **Discussion:** | Discussion: - The discussion remains excessively extensive and narrative in certain sections, particularly in explanations involving genetic mechanisms, developmental dental theories, and molecular influences, aspects that were not directly evaluated in the study; | We have carefully re-examined the Discussion and revised several sections to improve focus on the findings generated by the present study. In particular, portions discussing developmental dental theories, genetic influences, and molecular mechanisms have been condensed and streamlined. These topics are now included primarily to provide biological context for the observed morphometric patterns and are clearly presented as interpretations based on previously published literature rather than mechanisms directly evaluated in the present investigation. These revisions were undertaken to improve readability, objectivity, and alignment between the study objectives, results, and discussion. | Lines 403-409, lines 411-414, lines 420-422, lines 432-439,lines 443-445 |
| **Discussion:** | - Some interpretations still appear overly inferential in relation to the actual data obtained, especially regarding discriminative power, biological mechanisms, and possible clinical/forensic applications; | In response, we carefully reviewed the Discussion and Conclusion sections to ensure that all interpretations remain closely aligned with the evidence generated by the study. Statements relating to discriminative capability have been substantially tempered, particularly in light of the absence of cross-validation and the overlap observed in principal component analysis. Similarly, developmental and biological explanations have been reframed as possible interpretations supported by existing literature rather than direct conclusions of the present study. References to potential orthodontic and forensic applications have also been revised and are now presented as possible future research directions rather than established applications arising from the current findings. These modifications were made to improve scientific objectivity and ensure that the interpretation remains proportional to the data obtained. | Lines 413-415 (clinical)  Lines 420-422 (forensic)  Lines 443-445 (discriminative power related) |
| **Discussion:** | - The high discriminant accuracy observed should continue to be interpreted cautiously, considering the absence of cross-validation, the overlap observed in the PCA, and the lack of statistical significance in part of the results. | We agree that the discriminant function analysis results should be interpreted cautiously. In response, the manuscript has been revised to further reduce emphasis on the reported classification accuracy and to explicitly acknowledge the absence of cross-validation as a major limitation. Additional statements have been incorporated in the Results, Discussion, and Limitations sections emphasizing that the reported classification performance represents apparent in-sample results and should not be interpreted as evidence of generalizable predictive capability. We have also highlighted the substantial overlap observed in the principal component analysis and the fact that statistically significant shape differences were observed only for the maxillary second molar. These revisions were made to ensure that the interpretation of the discriminant analysis remains appropriately aligned with the evidence generated by the study. | Lines 443-445 |
| **Discussion:** | Limitations related to landmark reproducibility, intra/interexaminer reliability assessment, and external validation of discriminant models remain insufficiently addressed; | In response, the manuscript has been revised to provide a more detailed description of landmark reproducibility assessment. The Methods section now explicitly reports observer calibration procedures, intra- and inter-observer reliability assessment, repeated landmark digitization protocols, and global intraclass correlation coefficients (ICC), which ranged from 0.982 to 1.000, indicating excellent agreement. In addition, digitization error was evaluated using repeated landmark placement and a permutation-based nonparametric ANOVA, demonstrating that measurement error accounted for less than 10% of total variance.  Furthermore, the absence of cross-validation or external validation of the discriminant function analysis has now been more explicitly acknowledged as a limitation. The Discussion and Limitations sections have been revised to clarify that the reported classification accuracies represent apparent in-sample performance and may overestimate predictive capability in independent samples. These additions were made to improve methodological transparency and to provide a more balanced interpretation of the findings. | Lines 432 to 448 (limitations)  Lines 443-445 (for discriminant model) |
| **Discussion:** | - Some sections appear peripheral to the primary objective of the study and could be condensed to improve objectivity and readability of the discussion. | We have carefully reviewed the Discussion with particular attention to maintaining focus on the primary objectives and findings of the study. Sections considered peripheral to the main geometric morphometric findings have been condensed, and several explanatory passages have been streamlined to improve readability and objectivity. The revised Discussion places greater emphasis on the observed shape differences and allometric patterns, while limiting broader theoretical and applied considerations to brief contextual discussion where relevant. | Discussion |
|  | Conclusion: - The conclusion could briefly mention that the findings specifically refer to the studied population, reinforcing the limitation of generalizability already discussed previously and maintaining better coherence with the methodological limitations presented in the discussion. | We agree that the conclusion should explicitly reflect the population-specific nature of the findings and remain consistent with the limitations discussed in the manuscript. Accordingly, the Conclusion section has been revised to clarify that the observed patterns of sexual dimorphism were identified within the studied population and that caution should be exercised when extrapolating these findings to other populations. This modification improves coherence between the conclusions and the methodological limitations acknowledged in the Discussion. | Conclusion |
|  | General Suggestions:  Consider reporting more explicitly the landmark validation/reproducibility procedures in the main text (e.g., intraexaminer error, repeated landmark placement, or ICC), as this is particularly relevant in geometric morphometric studies; Interpretation of the discriminant function analysis (DFA) could be presented more conservatively, emphasizing that high classification accuracy does not necessarily imply high generalizable discriminative capability, especially in the absence of external validation or cross-validation; The discussion could be simplified by reducing lengthy theoretical sections related to biological/genetic mechanisms not directly evaluated in the study, prioritizing greater focus on findings actually produced by the morphometric analysis; Inclusion of a summarized methodological flowchart or analytical pipeline could improve clarity and reproducibility of the study, especially for readers less familiar with geometric morphometric analysis; A final revision of scientific language and textual fluency by a specialized editing service or native English reviewer is recommended to improve readability, objectivity, and overall manuscript quality. | The authors thank the reviewer for these constructive suggestions. Several of these recommendations have been incorporated during the revision process. The Methods section has been expanded to provide a more detailed description of observer calibration, intra- and inter-observer reliability assessment, repeated landmark digitization procedures, global ICC values, and measurement-error analysis. In addition, the interpretation of discriminant function analysis has been substantially revised to emphasize that the reported classification accuracies represent apparent in-sample performance and should not be interpreted as evidence of generalizable predictive capability in the absence of cross-validation or external validation.  The Discussion has also been carefully reviewed and condensed, with greater emphasis placed on the morphometric findings generated by the study and reduced focus on broader developmental, genetic, and theoretical considerations that were not directly investigated. Furthermore, the manuscript has undergone an additional review for scientific language, readability, consistency, and overall presentation.  We appreciate the suggestion regarding inclusion of a methodological flowchart. While we agree that such a figure may improve accessibility for readers unfamiliar with geometric morphometric analysis, the analytical workflow is described in detail within the Methods section and illustrated through the landmark configuration figures. Nevertheless, should the Editor consider a flowchart necessary, we would be pleased to incorporate one in a subsequent revision. |  |
|  | Grammatical:  Lines 62–63 (“holds immense potential” / “assisting prosthetic dentists in fabricating dental tooth surfaces”), | We thank the reviewer for this observation. The sentence has been revised to improve scientific objectivity, clarity, and academic style by avoiding promotional language and using more precise terminology. | Line 62 -65 |
|  | line 66 (“One well-known example”), | We thank the reviewer for this suggestion. The wording has been revised to improve academic style and maintain a more objective scientific tone. | Line 67-68 (regarding cope Osborne theory) |
|  | lines 82–84 (“derived from the ectoderm interact with the dental papilla”), | We thank the reviewer for identifying this issue. The sentence has been revised to improve grammatical accuracy, readability, and scientific clarity regarding the developmental interactions involved in tooth formation. | Line 83-86 |
|  | line 109 (“Presently, majority of the research”),  lines 109–110 (“focuses on size rather than shape of biological entities”), | We thank the reviewer for this observation. The sentence has been revised to improve grammatical accuracy and academic style. | Line 109-110 |
|  | lines 112–113 (“foundational basis for future applications”), | We thank the reviewer for this observation. The sentence has been revised to improve precision and academic style by removing redundant wording and adopting a more objective description of the study's contribution. | Line 112 |
|  | line 127 (“obtained prospectively from orthodontic patient records”), | We thank the reviewer for highlighting this ambiguity. The sentence has been revised to clarify the source and timing of data collection and to improve methodological transparency. | Line 128-129 |
|  | line 134 (“A detailed history of the demographics including the domicile/birthplace”), | We thank the reviewer for this observation. The sentence has been revised to improve grammatical accuracy and methodological clarity regarding the demographic information collected from participants. | Line 133-134 |
|  | line 136 (“born and brought up”), | We thank the reviewer for this suggestion. The wording has been revised to improve academic style and methodological precision. | Line 134-136 |
|  | line 159 (“improve robustness”), | We thank the reviewer for this observation. The wording has been revised to provide a more precise methodological justification for the final sample size | Line 155-161 |
|  | line 162 (“ensuring stability of the analysis”), | We thank the reviewer for this observation. The wording has been revised to avoid overstatement and to more accurately describe the relationship between sample size and the reliability of the morphometric analyses. | Line 160-161 |
|  | lines 175–176 (“provided a comprehensive framework”), | We thank the reviewer for this observation. The sentence has been revised to adopt a more objective and precise description of the role of the selected landmarks in the morphometric analysis. | Lines 177-178 |
|  | line 239 (“variation between the sex”), | We thank the reviewer for identifying this grammatical issue. The wording has been revised to improve clarity and scientific writing style. | Line 249 |
|  | line 245 (“significant association of size dependent shape change”), | We thank the reviewer for this observation. The sentence has been revised to improve grammatical clarity and to employ standard geometric morphometric terminology. | Lines 254-257 |
|  | line 252 (“for prediction of the shape”), | We thank the reviewer for this observation. The sentence has been revised to clarify that the regression analysis was used to evaluate the extent of allometric shape variation associated with centroid size rather than for predictive modeling. | Lines 254-257 |
|  | line 254 (“which indicate the mean difference”), and | We thank the reviewer for this observation. The sentence has been revised to provide a more accurate description of Procrustes and Mahalanobis distances within the context of geometric morphometric analysis. | Line 261-263 |
|  | lines 301–302 (“female teeth also were seen to show”) could be revised regarding methodological clarity, scientific objectivity, academic fluency, and grammatical/stylistic adequacy. | We thank the reviewer for this observation. The sentence has been revised to improve grammatical accuracy, readability, and scientific objectivity. | Line 313-315 |

# Reviewer 3

| **Editorial comment** | Comment | Reply | Location in manuscript (without track changes) |
| --- | --- | --- | --- |
| **Reviewer #3:** | This revised version is clearly improved. The title is now more appropriate, the conclusions are better aligned with the reported findings, landmark reliability has been added, bilateral handling is described, and the data availability statement is now repository-based. The core result is also presented more accurately: there is no significant sex-related shape difference for UM1, whereas UM2 shows a significant shape difference; allometry is significant in both teeth and larger in UM2. | The authors sincerely thank the reviewer for the positive assessment of the revised manuscript. We appreciate the reviewer’s recognition of the improvements made in response to the previous comments, including refinement of the title, clarification of the conclusions, inclusion of landmark reliability assessment, description of bilateral landmark handling, and provision of a repository-based data availability statement.  We are also grateful that the reviewer acknowledges the more accurate presentation and interpretation of the study findings, particularly regarding the absence of significant shape differences in the maxillary first molar, the presence of significant shape differences in the maxillary second molar, and the demonstration of significant allometry in both teeth with a greater contribution in the second molar. The reviewer’s constructive comments have substantially contributed to improving the clarity, methodological transparency, and scientific rigor of the manuscript. |  |
|  | That said, I do not think the manuscript is ready for acceptance yet. The main remaining issue is that the paper still gives too much weight to the discriminant/classification analysis. The abstract states that discriminant function analysis showed around 95% classification accuracy, but the methods make clear that this was calculated on the original dataset, without cross-validation, and the results also show substantial overlap in PCA and non-significant DFA p values. In this form, the classification result is not robust evidence of discriminatory performance and should not be highlighted in the abstract or interpreted as demonstrating practical sex-classification utility. It should be clearly framed as an apparent, in-sample result only. | In accordance with the reviewer's suggestion, the classification accuracy value has been removed from the Abstract. The Abstract now states only that discriminant function analysis was performed and that the findings should be interpreted cautiously because no cross-validation was undertaken. This revision avoids overemphasizing apparent in-sample classification performance and better reflects the exploratory nature of the analysis. | Line 48-50 |
|  | A second important issue is the treatment of bilateral data. The authors state that both right and left maxillary molars were included and that averaged landmark coordinates were used per individual. However, the manuscript does not explain whether left teeth were mirrored/reflected into a common orientation before averaging. In a 3D geometric morphometric workflow, this is methodologically important. Without a clear statement on mirroring and alignment of antimers, the averaging approach is difficult to evaluate. This point requires explicit clarification, and if mirroring was not performed, the analysis may need correction | The authors thank the reviewer for highlighting this important methodological aspect. We confirm that prior to averaging, the landmark configurations of the left maxillary molars were reflected (mirrored) into the orientation of the corresponding right molars to ensure homologous alignment of anatomical landmarks. Following reflection and alignment, landmark coordinates from the right and left sides were averaged to generate a single representative configuration for each participant. We agree that this procedure was not described explicitly in the original manuscript and have now revised the Methods section to clarify this step and improve reproducibility and methodological transparency. | Line 200-207 |
|  | The sample-size section is improved compared with the earlier version, but it still does not fully match the primary analysis. The calculation is based on previously reported linear mesiodistal tooth dimensions, whereas the study’s main endpoint is multivariate 3D shape. That may be acceptable as a pragmatic proxy in an exploratory study, but it should be presented as such and not as a firm justification that the sample is adequate for geometric morphometric inference. The sentence implying that the sample size “ensured stability of the analysis” is stronger than the methods support. | We thank the reviewer for this observation. We agree that the sample size calculation was based on previously reported linear odontometric measurements rather than geometric morphometric shape variables. As no established sample size estimation approach was available for the specific three-dimensional landmark-based analyses employed in this study, the odontometric data were used as a pragmatic guide for sample size determination. The manuscript has been revised to clarify this point and to avoid overstating the adequacy of the sample size for geometric morphometric inference. | Line 155-161 |
|  | The discussion remains better than before, but it still contains some overinterpretation and speculation.  In particular, the statement that the second molar “may be used with sufficient power to discriminate between sexes in the modern human” is not supported by the current analytical framework, especially given the lack of cross-validation and the overlap in PCA. | The authors thank the reviewer for this important observation and agree that the cited statement could be interpreted as overstating the discriminatory utility of the maxillary second molar. Given the absence of cross-validation, the overlap observed in principal component analysis, and the exploratory nature of the discriminant function analysis, the present study does not provide sufficient evidence to support claims regarding predictive sex-classification performance. Accordingly, the statement has been revised to focus on the observed shape differences and their potential biological relevance without implying practical discriminatory utility. | Discussion  Line 403-409  Line 420-422  Line 443-445 |
|  | Likewise, the broader orthodontic and forensic implications should be phrased much more cautiously unless the authors explicitly state that these are possible future directions rather than conclusions of the present study. The developmental/genetic explanations are plausible, but they remain interpretive rather than directly tested here and should be framed accordingly. | The authors thank the reviewer for this important observation and agree that the broader orthodontic and forensic implications discussed in the manuscript extend beyond the direct scope of the present study. Similarly, the developmental and genetic mechanisms referenced in the discussion are based on previously published literature and were not directly evaluated in the present investigation.  Accordingly, the Discussion and Conclusion sections have been revised to clearly distinguish between the findings generated by the study and their possible biological interpretations. Statements regarding developmental and genetic influences have been rephrased as potential explanations for the observed patterns of shape variation. In addition, references to orthodontic and forensic applications have been presented as possible future research directions rather than conclusions directly supported by the present data. |  |
|  | The added reliability analysis is a useful improvement, but the reporting is still somewhat incomplete. The manuscript reports ICC values from 0.982 to 1.000 and states that digitization error accounted for less than 10% of variance, but it does not fully explain whether the ICCs were calculated per landmark, per coordinate set, or as a global summary. The nonparametric ANOVA-based digitization-error analysis also needs slightly clearer description so readers can understand exactly what was repeated and how error was partitioned. | We agree that additional clarification regarding the reliability assessment would improve methodological transparency. The ICC values reported in the manuscript represent global ICC estimates calculated from the complete landmark coordinate datasets obtained during repeated landmark digitization rather than individual landmark-specific ICCs. To address this point, the Methods section has been revised to explicitly describe the basis of ICC calculation, the protocol used for intra- and inter-observer reliability assessment, and the subset of models included in the analysis.  In addition, the description of the digitization-error assessment has been expanded to clarify that repeated landmark digitization was performed on a subset of dental models and that a one-way nonparametric ANOVA with a randomized permutation procedure (10,000 iterations) was used to partition total variance into biological variation and measurement error components. These revisions provide a clearer explanation of the reliability and measurement-error analyses performed in the study. | Lines 209-224 |
|  | There are also some smaller issues of wording and presentation. The phrasing “pretreatment dental casts were obtained prospectively from orthodontic patient records” is awkward and should be clarified. The age distribution is now reported, which is helpful, but the modest age difference between males and females should at least be acknowledged, even if the authors believe it is unlikely to affect crown morphology materially in this young sample. The language overall is much improved, but the manuscript would still benefit from another careful stylistic edit. | The wording describing the source of the dental casts has been revised to improve methodological clarity. In addition, we agree that the observed age difference between male and female participants should be acknowledged. Although crown morphology is established prior to the age range represented in the present sample and is therefore unlikely to be materially influenced by this difference, a statement acknowledging this potential limitation has now been added to the manuscript.  Furthermore, the manuscript has undergone an additional review for language, style, and readability to improve overall clarity and consistency. | Lines 128-129 |
|  | In summary, this revision is substantially better and closer to publishable form. The descriptive morphometric finding for UM2 is potentially publishable. However, the bilateral-data workflow must be clarified, the classification analysis must be substantially toned down, and the discussion should be further restrained. If those points are addressed carefully, I think the manuscript could become suitable for publication. | The authors sincerely thank the reviewer for the constructive evaluation of the revised manuscript and for recognizing the substantial improvements made during the revision process. We appreciate the positive assessment of the principal morphometric findings, particularly regarding the maxillary second molar.  In response to the remaining concerns, we have further revised the manuscript to improve methodological transparency and ensure that the interpretation of findings remains fully aligned with the evidence presented. Specifically, the bilateral-data workflow has been clarified by explicitly describing the reflection (mirroring) and averaging procedures applied to antimeric teeth. The emphasis on discriminant function analysis and classification performance has been substantially reduced, including revision of the Abstract and Discussion to avoid implying predictive utility in the absence of cross-validation. In addition, the Discussion and Conclusion have been further refined to distinguish observed findings from literature-based interpretations and to present potential orthodontic, forensic, developmental, and genetic implications as hypotheses or future research directions rather than direct conclusions of the present study.  We are grateful for the reviewer’s detailed comments, which have helped improve the clarity, methodological rigor, and overall presentation of the manuscript. |  |

# Reviewer 5

| Editorial comment | Comment | Reply | Location in manuscript (without track changes) |
| --- | --- | --- | --- |
| Reviewer #5: | The authors have taken my comments into account. Following the revisions, the quality of the article has improved significantly. | The authors sincerely thank the reviewer for the positive assessment of the revised manuscript and for recognizing the improvements made following the previous round of review. We appreciate the careful attention to the remaining editorial and formatting issues. |  |
|  | I would recommend checking the manuscript once more for still left proofreading errors: for example,  removing text highlighting (lines 48, 49, 73),  standardize the font size (lines 184, 306),  add missing periods at the end of sentences (lines 215, 442),  separate words (lines 167, 170), and  check the use of capital letters in Table 1 (landmarks 13, 15, 16). | In response to the reviewer’s suggestions, the manuscript has been thoroughly proofread and the identified issues have been corrected. Text highlighting has been removed, font formatting has been standardized, punctuation and spacing errors have been corrected, and capitalization in Table 1 has been reviewed and revised for consistency. In addition, the manuscript has undergone a further review for language, formatting, and typographical accuracy throughout. | Lines as mentioned in the reviewers comments is corrected |
