# Supplementary material for: Geometric morphometric analysis of maxillary molar sexual dimorphism: A cross-sectional study
Source: PLoS One. 2026 Aug 7;21(8):e0355612. doi: 10.1371/journal.pone.0355612 (PMC13450795; doi:10.1371/journal.pone.0355612)
Supplement: Supplementary file 3 [file pone.0355612.s004.docx]

| Reviewer | Comment | Response | Location in manuscript |
| --- | --- | --- | --- |
| Reviewer #1: | With the help of the Reviewers, this submitted draft could be considerably improved. Notwithstanding, several further revisions would seem mandatory, please see below. | We sincerely thank the Reviewer for the careful evaluation of our manuscript and for the constructive comments and suggestions. We have carefully considered each point and have revised the manuscript accordingly. We believe these revisions have substantially improved the clarity, methodological rigor, and overall quality of the manuscript. Our detailed point-by-point responses to each comment are provided below. |  |
| Abstract | - "(...), explained by the concept in literature of farther distance from the primary field of influence and genetic associations." This is not considered a conclusion from your study. - With your Conclusions, do not simply repeat your outcome. Do not speculate. Stick exclusively to your aims, and provide generalizing responses. | We thank the Reviewer for this valuable observation. The conclusion of the abstract has been revised to avoid speculative interpretation regarding the developmental field theory and genetic associations, as these were discussed only as possible explanations in the Discussion and were not directly investigated in the present study. The revised conclusion now focuses on the study's primary findings in relation to its objective and provides a concise, evidence-based summary without repeating the results or extending beyond the data. |  |
| Intro | - "Tooth size is influenced by a variety of (...)." Remember that one sentence does not constitute one paragraph. Provide digestible units. | We thank the Reviewer for this helpful suggestion. The Introduction has been revised to improve paragraph structure and readability. The single-sentence paragraph has been merged with the adjacent text to form a coherent paragraph while preserving the intended flow of information. |  |
| Methods | - "(...) and with the 1964 Helsinki Declaration and its later amendments or comparable ethical standards." Reference missing. | We thank the Reviewer for this observation. An appropriate reference to the World Medical Association Declaration of Helsinki has now been added in the Methods section to support the ethical statement. |  |
|  | - "This study was not preregistered as it was an observational cross-sectional study using existing clinical records." Remember that "existing clinical records" would not seem a sound reason to NOT pre-register. The latter refers to several other reasons. Please revise carefully | We thank the Reviewer for this important observation. We agree that the use of existing clinical records is not, by itself, a justification for the absence of preregistration. The statement has been revised to clarify that preregistration was not performed because the study was an observational cross-sectional study initiated before preregistration was adopted as a routine requirement for such studies at our institution. This revision more accurately reflects the study context without implying that the use of existing clinical records precludes preregistration. |  |
|  | - "(...) cannot be assumed.." - revise for typos.  - Same with "(...) was not analysed separately. ." Revise carefully. | We thank the Reviewer for identifying these typographical errors. The manuscript has been carefully revised to correct the punctuation and improve overall readability. |  |
|  | - Please revise carefully for uniform expressions. Compare "distolingual/mesiolingual" and "distopalatal/mesiopalatal". | We thank the Reviewer for this helpful observation. The manuscript has been carefully revised to ensure consistent anatomical terminology throughout. References to the palatal aspects of the maxillary molars have been standardized, and uniform terminology has been used throughout the text, tables, and figure legends. |  |
|  | - "The null hypothesis tested was that there is no significant difference in shape or size of maxillary molars between males and females." H0 already has been provided with the Intro section. Why do you repeat this H0 here? | We thank the Reviewer for this observation. We agree that the null hypothesis was already clearly stated at the end of the Introduction. To avoid unnecessary repetition, the statement has been removed from the Methods section. |  |
| Results | - "mean age of 18.62 years (SD = 2.49)" - this would seem spuriously accurate. One digit would seem sufficient. Revise carefully. | We thank the Reviewer for this helpful suggestion. The demographic data have been revised to report age and standard deviation to one decimal place, thereby avoiding unnecessary precision and improving consistency with standard reporting practices. |  |
|  | - Again, revise for sound full stop use, see "(...) (p ≤ 0.001). (Table 3) ." | We thank the Reviewer for this careful observation. The punctuation in the cited sentence has been corrected by removing the unnecessary full stops and ensuring appropriate placement of the table citation. In addition, the entire manuscript has been carefully reviewed for similar punctuation and formatting inconsistencies, and all identified instances have been corrected to ensure uniformity and improve readability. |  |
|  | - Same with "(...) based on shape variables. [3]." Revise carefully. | We thank the Reviewer for this observation. The punctuation has been corrected by integrating the citation appropriately within the sentence. In addition, the manuscript has been carefully reviewed for similar punctuation inconsistencies, and all identified instances have been revised to ensure uniform formatting. |  |
|  | - Why do you stick to "≤"? See "(p ≤ 0.001)". | We thank the Reviewer for this observation. The statistical reporting has been revised to conform to conventional biomedical journal style. The expression "p ≤ 0.001" has been replaced with "p < 0.001". |  |
| Discussion | - "The null hypothesis stated that there is no significant difference in the shape or size of maxillary permanent molars between males and females." See comments on repetition of H0 given above, and revise carefully. | We thank the Reviewer for this observation. We agree that restating the null hypothesis in the Discussion is unnecessary, as it has already been presented in the Introduction. The Discussion has therefore been revised to avoid repetition and to directly interpret the study findings. |  |
|  | - Again, revise for uniform formatting, see "p = 0.01" and "p= 0.150". | We thank the Reviewer for this observation. The formatting of p-values has been standardized throughout the manuscript to ensure consistency with the journal's style. Appropriate spacing around statistical symbols has been applied, and all p-values have been reviewed for uniform presentation. |  |
|  | - "The shape of the molar has been correlated with craniofacial complexes by Polychronis and Halazonetis (2014)." Reference number is missing. | We thank the Reviewer for this observation. The appropriate reference citation has now been added to the first sentence of the paragraph to ensure that all statements are adequately referenced. |  |
|  | - Again, please compare "distopalatal cusp" and "mesiolingual cusp". Revise carefully for uniform terms and descriptions. | We thank the Reviewer for this helpful observation. The manuscript has been carefully revised to ensure consistent anatomical terminology throughout. References to the palatal aspects of the maxillary molars have been standardized by using "palatal" terminology consistently (e.g., mesiopalatal and distopalatal) where anatomically appropriate, while retaining established odontometric terms such as "buccolingual width." |  |
|  | - Again, revise for typos, see "(...) and dental morphology. [32,34,35]." | We thank the Reviewer for identifying this typographical error. The punctuation has been corrected in the cited sentence. In addition, the manuscript has been carefully reviewed for similar punctuation inconsistencies, and all identified instances have been revised to ensure uniform formatting throughout. |  |
|  | - Same with "area-based and Procrustes-based approaches". | We thank the Reviewer for identifying this formatting inconsistency. The punctuation has been corrected in the cited sentence. In addition, the manuscript has been carefully reviewed to identify and correct similar punctuation and formatting inconsistencies throughout, ensuring uniform presentation. |  |
|  | - Revise for uniform citation style. See "Morita W et al. (2016)", and compare to "Popovici et al (2022)". Revise carefully (as a general recommendation, please reduce Authors' names with your text; instead, stick to your main thoughts). | We thank the Reviewer for this valuable suggestion. The manuscript has been revised to ensure a uniform citation style throughout. In addition, where appropriate, narrative references to individual authors have been reduced and replaced with neutral statements supported by reference citations, thereby improving readability and maintaining consistency with the journal's Vancouver referencing style. | Spain  Romania  Metamerism |
|  | - "This is consistent with the study done by Morita W et al. (2016), who studied the metameric variation of human molars." Again, reference number is missing. | We thank the Reviewer for this observation. The sentence has been revised to improve consistency with the citation style used throughout the manuscript, and the appropriate reference citation has been added. |  |
| Figs | - Figures as attached to this manuscript would not seem reproducible. Please double check, and ensure to provide high-quality images. |  |  |
| Reviewer #3: Recommendation: Minor Revision | This version is substantially improved and much closer to publication. The main conclusions are now better aligned with the data: the manuscript clearly states that shape differences were significant only for UM2, not UM1; the abstract and discussion now acknowledge that the classification findings should be interpreted cautiously because no cross-validation was performed; the bilateral workflow is clarified by stating that left teeth were mirrored before averaging; the reliability section is more complete; and the sample-size justification is presented more honestly as a pragmatic proxy based on prior linear odontometric data. | We sincerely thank the Reviewer for the encouraging comments and for recognizing the improvements made to the manuscript. We are pleased that the revisions have addressed the major concerns regarding the interpretation of the findings, methodological clarity, reliability assessment, and sample size justification. We appreciate the constructive feedback, which has helped strengthen the scientific quality and transparency of our work. |  |
|  | The remaining issues are now mostly about emphasis and presentation rather than fundamental methodological flaws. The main one is that the manuscript still gives quite a lot of prominence to the discriminant analysis: the Results section still reports ~95% apparent accuracy, Table 4 still centers those numbers, and Figure 4 still visually highlights “Accuracy 95%,” even though the manuscript also states that these are in-sample results without cross-validation and that PCA shows substantial overlap with non-significant DFA p values. I suggest further toning this down in the main text, or moving part of it to supplementary material (Figure 4), so readers do not overinterpret exploratory separation as validated classification performance. | We sincerely thank the Reviewer for this valuable suggestion. We agree that the discriminant function analysis should be presented as an exploratory assessment rather than as validated classification performance. Accordingly, the Results section has been revised to further reduce the emphasis on the apparent classification accuracy and to reinforce that these findings represent in-sample exploratory results without cross-validation. In addition, the title and legend of Table 4 have been revised to clarify the exploratory nature of the analysis. Figure 4 has also been modified by removing the graphical display of classification accuracy, thereby focusing the figure on the relative landmark displacements and morphological differences between the sexes. These revisions are intended to ensure that the presentation of the results is appropriately balanced and consistent with the study's objectives and methodological limitations. |  |
|  | The discussion is improved and is generally more restrained, but the conclusion still edges slightly toward application. The final sentence says that the developmental and applied implications warrant future investigation for clinical applications in orthodontics and forensic odontology. That is acceptable only if framed strictly as future work; it should not read as an implication already supported by this study. I would recommend softening this final sentence further. | We thank the Reviewer for this constructive suggestion. The concluding statement has been revised to further emphasize that any potential applications in orthodontics and forensic odontology remain speculative and require confirmation through future research. The conclusion has been modified to ensure that it reflects only the findings of the present study and appropriately frames any broader applications as areas for future investigation. |  |
|  | There are also some residual editorial issues. The manuscript is much cleaner than before, but minor redundancies, small grammatical problems, and a few awkward sentences remain, especially in the Introduction and Discussion. These are readily fixable in a final revision. | We sincerely thank the Reviewer for this valuable observation and for recognizing the improvements made to the manuscript. In response to this suggestion, the manuscript has undergone a further comprehensive editorial revision to improve clarity, readability, and consistency. Minor redundancies, grammatical errors, and awkward sentence constructions, particularly in the Introduction and Discussion sections, have been corrected, and the manuscript has been carefully proofread to ensure a clear and concise presentation. |  |
|  | Overall, I think the authors have addressed the main scientific concerns from prior rounds. At this stage, I would support publication after minor revision, mainly to further de-emphasize the unvalidated classification results, slightly tighten the conclusion, and complete a final language edit. | We sincerely thank the Reviewer for the careful evaluation of our manuscript and for the constructive comments provided throughout the review process. We appreciate the recognition that the major scientific concerns have been satisfactorily addressed. In accordance with the Reviewer's recommendations, we have further de-emphasized the exploratory discriminant function analysis by revising the relevant text, table, and figure, refined the conclusion to more closely reflect the findings of the present study, and performed a final comprehensive language and editorial review of the manuscript. We believe that these revisions have further improved the clarity, balance, and overall quality of the manuscript. |  |
| Reviewer #5: | The authors have substantially improved their work. All of my comments have been addressed. The paper is now acceptable for publication. | We sincerely thank the Reviewer for the careful evaluation of our manuscript and for the constructive comments provided throughout the review process. We greatly appreciate your positive assessment and are pleased that the revisions have satisfactorily addressed all of your concerns. Your insightful feedback has significantly contributed to improving the quality and clarity of the manuscript. |  |
