# Supplementary material for: Geometric morphometric analysis of maxillary molar sexual dimorphism: A cross-sectional study
Source: PLoS One. 2026 Aug 7;21(8):e0355612. doi: 10.1371/journal.pone.0355612 (PMC13450795; doi:10.1371/journal.pone.0355612)
Supplement: Peer review history

# Author comment: Author response to Decision Letter 0

**DOI:** 10.1371/journal.pone.0355612.r001

**Transfer Alert**

This paper was transferred from another journal. As a result, its full editorial history (including decision letters, peer reviews and author responses) may not be present.

6 Nov 2025

# Peer review: Decision Letter 0

**DOI:** 10.1371/journal.pone.0355612.r002

**Authors:** Andrej Kielbassa

5 Apr 2026

PONE-D-25-59851

Geomorphometric Insights into Maxillary Molar Dimorphism: Shape as a Tool for Clinical Applications

PLOS One

Dear Dr. Ahmed,

Thank you for submitting your manuscript to PLOS ONE. After careful consideration, we feel that it has merit but does not fully meet PLOS ONE’s publication criteria as it currently stands. Therefore, we invite you to submit a revised version of the manuscript that addresses the points raised during the review process.

- Please note that, while differing with their recommendations to some some extent, our external reviewers have finally addressed several shortcomings and drawbacks, and the latter have been indicated.
- I have additionally double checked your submitted draft, to come to a more balanced decision. Specific feedback from my evaluation of the manuscript has been added below. You surely will agree that your manuscript is not considered ready to proceed.
- Please note that I have decided to give you the opportunity to carefully but thoroughly revise your draft. You should bear in mind that our final decision will depend on the quality of your revised draft, and that one revision is considered standard. Remember that re-review of your re-submitted draft is considered mandatory.

-->Please submit your revised manuscript by May 20 2026 11:59PM. If you will need more time than this to complete your revisions, please reply to this message or contact the journal office at plosone@plos.org. When you're ready to submit your revision, log on to https://www.editorialmanager.com/pone/ and select the 'Submissions Needing Revision' folder to locate your manuscript file.

Please include the following items when submitting your revised manuscript:

- A letter that responds to each point raised by the academic editor and reviewer(s). You should upload this letter as a separate file labeled 'Response to Reviewers'.
- A marked-up copy of your manuscript that highlights changes made to the original version. You should upload this as a separate file labeled 'Revised Manuscript with Track Changes'.
- An unmarked version of your revised paper without tracked changes. You should upload this as a separate file labeled 'Manuscript'.

If you would like to make changes to your financial disclosure, please include your updated statement in your cover letter. Guidelines for resubmitting your figure files are available below the reviewer comments at the end of this letter.

If applicable, we recommend that you deposit your laboratory protocols in protocols.io to enhance the reproducibility of your results. Protocols.io assigns your protocol its own identifier (DOI) so that it can be cited independently in the future. For instructions see: https://journals.plos.org/plosone/s/submission-guidelines#loc-laboratory-protocols. Additionally, PLOS ONE offers an option for publishing peer-reviewed Lab Protocol articles, which describe protocols hosted on protocols.io. Read more information on sharing protocols at https://plos.org/protocols?utm_medium=editorial-email&utm_source=authorletters&utm_campaign=protocols.

As the corresponding author, your ORCID iD is verified in the submission system and will appear in the published article. PLOS supports the use of ORCID, and we encourage all coauthors to register for an ORCID iD and use it as well. Please encourage your coauthors to verify their ORCID iD within the submission system before final acceptance, as unverified ORCID iDs will not appear in the published article. *Only*  the individual author can complete the verification step; PLOS staff *cannot*  verify ORCID iDs on behalf of authors.

We look forward to receiving your revised manuscript.

Kind regards,

Andrej M Kielbassa

Academic Editor

PLOS One

Journal Requirements:

When submitting your revision, we need you to address these additional requirements.

1. Please ensure that your manuscript meets PLOS ONE's style requirements, including those for file naming. The PLOS ONE style templates can be found at

https://journals.plos.org/plosone/s/file?id=wjVg/PLOSOne_formatting_sample_main_body.pdf and

https://journals.plos.org/plosone/s/file?id=ba62/PLOSOne_formatting_sample_title_authors_affiliations.pdf

2. Thank you for stating the following in the Acknowledgments Section of your manuscript:

“Study supported in part by a the Science and Engineering Research Board (SERB), Department of Science and Technology, Government of India (file number CRG/2020/001057)”

We note that you have provided funding information that is not currently declared in your Funding Statement. However, funding information should not appear in the Acknowledgments section or other areas of your manuscript. We will only publish funding information present in the Funding Statement section of the online submission form.

Please remove any funding-related text from the manuscript and let us know how you would like to update your Funding Statement. Currently, your Funding Statement reads as follows:

“Study supported in part by a the Science and Engineering Research Board (SERB), Department of Science and Technology, Government of India (file number CRG/2020/001057)”

Please include your amended statements within your cover letter; we will change the online submission form on your behalf.

3. We note that the grant information you provided in the ‘Funding Information’ and ‘Financial Disclosure’ sections do not match.

When you resubmit, please ensure that you provide the correct grant numbers for the awards you received for your study in the ‘Funding Information’ section.

4. Thank you for stating the following financial disclosure:

“Study supported in part by a the Science and Engineering Research Board (SERB), Department of Science and Technology, Government of India (file number CRG/2020/001057)”

Please state what role the funders took in the study.  If the funders had no role, please state: "The funders had no role in study design, data collection and analysis, decision to publish, or preparation of the manuscript."

If this statement is not correct you must amend it as needed.

Please include this amended Role of Funder statement in your cover letter; we will change the online submission form on your behalf.

5. In the online submission form you indicate that your data is not available for proprietary reasons and have provided a contact point for accessing this data. Please note that your current contact point is a co-author on this manuscript. According to our Data Policy, the contact point must not be an author on the manuscript and must be an institutional contact, ideally not an individual. Please revise your data statement to a non-author institutional point of contact, such as a data access or ethics committee, and send this to us via return email. Please also include contact information for the third party organization, and please include the full citation of where the data can be found.

6. Your ethics statement should only appear in the Methods section of your manuscript. If your ethics statement is written in any section besides the Methods, please delete it from any other section.

7. Please include a separate caption for each figure in your manuscript.

8. If the reviewer comments include a recommendation to cite specific previously published works, please review and evaluate these publications to determine whether they are relevant and should be cited. There is no requirement to cite these works unless the editor has indicated otherwise.

[Note: HTML markup is below. Please do not edit.]

Reviewers' comments:

Reviewer's Responses to Questions

**Comments to the Author**

1. Is the manuscript technically sound, and do the data support the conclusions?

The manuscript must describe a technically sound piece of scientific research with data that supports the conclusions. Experiments must have been conducted rigorously, with appropriate controls, replication, and sample sizes. The conclusions must be drawn appropriately based on the data presented.

Reviewer #1: No

Reviewer #2: Partly

Reviewer #3: Partly

Reviewer #4: No

Reviewer #5: Yes

Reviewer #6: No

**********

2. Has the statistical analysis been performed appropriately and rigorously?

Reviewer #1: No

Reviewer #2: N/A

Reviewer #3: No

Reviewer #4: No

Reviewer #5: Yes

Reviewer #6: No

**********

3. Have the authors made all data underlying the findings in their manuscript fully available?

The PLOS Data policy requires authors to make all data underlying the findings described in their manuscript fully available without restriction, with rare exception (please refer to the Data Availability Statement in the manuscript PDF file). The data should be provided as part of the manuscript or its supporting information, or deposited to a public repository. For example, in addition to summary statistics, the data points behind means, medians and variance measures should be available. If there are restrictions on publicly sharing data—e.g. participant privacy or use of data from a third party—those must be specified.

Reviewer #1: No

Reviewer #2: No

Reviewer #3: No

Reviewer #4: No

Reviewer #5: Yes

Reviewer #6: No

**********

4. Is the manuscript presented in an intelligible fashion and written in standard English?

PLOS ONE does not copyedit accepted manuscripts, so the language in submitted articles must be clear, correct, and unambiguous. Any typographical or grammatical errors should be corrected at revision, so please note any specific errors here.

Reviewer #1: No

Reviewer #2: No

Reviewer #3: No

Reviewer #4: Yes

Reviewer #5: Yes

Reviewer #6: No

**********

5. Review Comments to the Author

Please use the space provided to explain your answers to the questions above. You may also include additional comments for the author, including concerns about dual publication, research ethics, or publication ethics. (Please upload your review as an attachment if it exceeds 20,000 characters)

Reviewer #1: Title

- Please add type of study. No capital letters, please consult some recently published Plos One papers.

- "What do you mean when referring to "Shape as a Tool for Clinical Applications". I do not see any connections in the form of a "Tool for Clinical Applications". Please clarify.

Abstract

- Please provide maximum information here. See Journal guidelines, the maximal word count is 300. Go to https://journals.plos.org/plosone/s/submission-guidelines, and revise carefully.

- You aimed to "investigate the geometric morphometric shape dimorphisms of the Permanent Maxillary First (UM1) and Second Molars (UM2)". Please stick to these aims, both with your results and with your conclusions.

- With regard to "(P<0.01)", please stick to Journal style.

- "Cognizance of the shape changes, asymmetry and allometry in molars is important for anthropological, developmental, clinical and forensic applications." Did you investigate "anthropological, developmental, clinical, or forensic applications"? I guess you did not.

- With your Conclusions, please stick exclusively to your revised/adapted aims, and do not overstate. Do not simply repeat your results here. Do not speculate. Do not provide well-accepted (but unambiguous or even meaningless) phrases. Instead, provide a reasonable and generalizable extension of your outcome.

- This section has not been convincingly elaborated.

Intro

- "(...) but also enhance esthetics. [1]" Again, stick to Journal style, and consult some recently published Plos One papers. Citation style would be "(...) but also enhance esthetics [1]." Revise thoroughly.

- "Interestingly, males and females have different patterns of distribution of Amelogenin, an enamel protein." With each and every fact, please provide sound references.

- "It is established that the X chromosome determines enamel thickness, whereas the Y chromosome influences both enamel and dentin thickness. [8]" What about females with XX chromosomes, having no Y chromosome? In these cases, dentin thickness will not be influenced?

- Neither aims nor objectives have been convincingly elaborated. Please provide a sound rationale.

- "The objective of the present study is to explore the relationship between shape and size differences in tooth crowns, focusing on the hypothesis that males, due to the presence of both X and Y chromosomes, exhibit greater enamel and dentin thickness, thereby affecting the shape of the tooth crown more significantly." First, this is not a study proposal, please switch to past tense. Second, please provide a sound NULL HYPOTHESIS.

- Same with "We hypothesize that molar shape, as assessed by landmark data in three dimensions, exhibits characteristic features of sexual dimorphism." Again, switch to past tense. Why do you repeat your information here?

- Same with "The present study aims to determine the sexual dimorphism and discriminating capabilities of both shape and size of the maxillary permanent molars." Please revise carefully.

Meths

- Please double check these sentence: "Abe K et al. (1996) reported the sexual dimorphism in the size of the human molars. [11] The upper first molars reported significantly larger sizes in males (first molar: 10.57±0.52; second molar: 9.98±0.58) compared to the females (first molar: 10.15±0.55; second molar: 9.47±0.60)." First, this section refers to methodology, so please do not mix with aspects sticking to a literature review. Second, what does "10.57±0.52" mean? Revise carefully.

- Again, always stick to sound references, see "In 3D geometric morphometry, it is standard practice to ensure that the sample size is at least three times the number of variables being analyzed."

- You sample size calculation would seem hard to follow. You have stated that "Based on the centroid values of males and females of the upper first premolar and upper second premolar", and it remains unclear why you have calculated with premolar values when going for a study on molars.

- What about the pre-registration date and number of your study?

- With ALL materials and methodologies (including statistical software), please use general/non-proprietary names with your text, followed by (brand name; manufacturer, city, ST[ate - abbreviated, if US], country) in parentheses. Stick to semicolon. Please ensure reproducibility, and revise thoroughly.

- Aspects referring to your statistical analysis would seem missing. Remember that you have provided p values, and you have started with H0.

Results

- "(...) but not in first molar (P value 0.1498). (Table 2)" Revise for sound full stops.

- "P" would seem clear and self-explanatory. Do not use "P value".

- Please compare "(P value 0.1498)" and "(p =0.0012)", and revise for sound and uniform Journal style. Stick to 3-digit p values.

- Again, what does "The procrustes distances were 0.0229 and 0.0284" mean?

- Another example would be "The distal marginal ridge is narrower in females (landmarks 11,27) and mesial marginal ridge was narrower in males (landmarks 5,21)." What does this mean? What is meant when referring to "narrower"? Do you refer to "significant differences" here?

- Same with expressions like "more buccally placed", "more lingually placed", or "more cervically placed".

- Please note that the aspects given above would not seem an exhaustive list. Revise carefully, but thoroughly.

Disc

- What about H0?

- "Butler’s field model (1939) and Osborn’s Clone model (1978) are the two theories that describe tooth formation." Reference numbers missing.

- Same with "A study by Batbayar et al. (2016), showed that in the absence of the maxillary first molar the second molar size is larger than the group of individuals with the presence of first molar." Please remember that "This manuscript is an updated submission of our work previously prepared for PLOS Computational Biology, now revised and formatted in accordance with the guidelines of PLOS ONE." Consequently, a sound piece of work would seem expectable, you surely will agree.

- Same with "Morita et al. (2014) have shown that the hypocone has higher rates of variability as compared to (...)." Reference number missing.

- Same with "Developmental analysis showed that crown size is dependent on (...)" and with "If the duration of the cusp development takes longer time it does so at the expense of the later forming cusps." There are many mor references missing with your thoughts given with this section.

- "(...) there are studies, showing lack of sexual dimorphism of molars." Do you agree that the word studieS would call for referenceS?

- Again, revise for sound full stop and spacebar use, see "(...) of Romania. [22]The period of (...)".

- What is the "mesiolingual cusp tips in maxillary second molar"?

- "The findings presented in this manuscript were also discussed at the 19th National Conference of The Indian Association of Forensic Odontology, conducted by G Pulla Reddy Dental College and Hospital, Kurnool, Andhra Pradesh." Would this be of any relevance here?

- What about the strengths of your study?

- There would seem more limitations to be discussed.

- What about the responses to your teaching objectives?

Concl

- Again, with your Conclusions, please stick exclusively to your revised/adapted aims, and do not overstate. Do not simply repeat your results here. Do not speculate. Do not provide well-accepted (but unambiguous or even meaningless) phrases. Instead, provide a reasonable and generalizable extension of your outcome.

- "In summary, the use of 3D shape coordinates provides a more comprehensive understanding of tooth morphology, with significant implications for dental restoration, orthodontics, and forensic identification." This has not been investigated, has it?

Refs

- Revise for sound and uniform Journal style.

Figs/Tables

- Please revise for sound legends. The latter must be self-explanatory.

In total, this revised and re-submitted draft would seem to suffer from numerous shortcomings. Please note that it would seem clear (and your surely will confirm) that all 6 Co-Authors have "read, reviewed, revised, and approved" this submitted draft. Consequently, a faultless draft would seem expectable. Moreover, some major drawbacks do not allow for proceeding with this submission.

Reviewer #2: Review Comments to the Author

The manuscript explores an interesting and relevant topic, investigating sexual dimorphism in maxillary molars using 3D geometric morphometric analysis. The methodological approach is appropriate, and the use of landmark-based morphometry represents a strength of the study. However, several important issues need to be addressed to improve the scientific rigor, clarity, and overall impact of the manuscript.

First, there is a noticeable mismatch between the title and the content of the study. While the title emphasizes clinical applicability, the manuscript provides limited discussion on how the findings can be translated into clinical practice. The authors are encouraged to expand the discussion by including concrete clinical scenarios (e.g., implications for orthodontic planning, restorative procedures, or occlusal reconstruction). Alternatively, the title should be revised to better reflect the actual scope of the study.

Second, key sample characteristics are missing. The manuscript does not report the age distribution of the participants, which is a relevant variable in dental morphology due to potential effects of wear and age-related changes. This information should be included and discussed. Additionally, the study does not specify whether right or left molars were analyzed, nor whether bilateral symmetry was considered. Given that asymmetry is mentioned, this clarification is essential.

Third, the rationale for selecting only maxillary molars is not clearly justified. The authors should provide a scientific basis for this choice.

Regarding methodology, although appropriate statistical approaches appear to have been used (e.g., Procrustes analysis, PCA, discriminant function analysis, and regression), the reporting is insufficient. Statistical tests such as ANOVA and regression should be clearly described in the Methods section, including assumptions, significance levels, and analytical procedures. Additionally, no information is provided regarding intra- or inter-observer reliability for landmark placement, which is critical in geometric morphometric studies.

The sample size calculation is presented; however, there are inconsistencies in the number of landmarks and variables considered (28, 18, and 16), which should be clarified. The use of convenience sampling should also be acknowledged as a limitation.

Another important concern relates to the Discussion section. Several statements are presented without appropriate references, using expressions such as “many studies have shown” or “it is known that.” All scientific claims, particularly those related to genetic influence, tooth development, and sexual dimorphism, should be supported by relevant and preferably recent literature.

Finally, the manuscript does not clearly ensure compliance with data availability requirements. The raw data underlying the analyses (e.g., landmark coordinates) are not explicitly provided or linked to a public repository. This should be addressed in accordance with journal policies.

In terms of language, the manuscript is generally understandable but would benefit from careful revision to improve clarity, grammar, and consistency. Some sentences are overly complex, and minor typographical and organizational issues are present.

In conclusion, the study has merit, but substantial revisions are required to improve methodological transparency, strengthen the discussion, and align the manuscript with its stated clinical relevance.

Reviewer #3: The manuscript addresses a relevant question and applies a potentially useful 3D geometric morphometric approach to maxillary molar sexual dimorphism. The study is of interest, but the manuscript requires substantial revision before it can be considered further.

First, the conclusions overstate the results. The manuscript concludes that both UM1 and UM2 can discriminate sex, but the reported analyses show a significant shape difference only for UM2, not UM1. This inconsistency appears in the abstract, results, and conclusion, and the claims should be revised to match the actual findings.

Second, the discrimination analysis needs much clearer reporting. The manuscript reports about 95% classification accuracy, yet Table 4 shows non-significant P values, and the PCA is described as showing a homogeneous distribution of male and female samples. The authors should explain exactly how classification accuracy was obtained, and whether any cross-validation or independent validation was performed. Without that, the forensic and clinical claims are too strong.

Third, landmark reliability is not reported. Because the study depends on 28 manually placed 3D landmarks, intra-observer reproducibility, and ideally inter-observer agreement or digitization error, should be presented. This is important for judging the robustness of the morphometric findings.

Fourth, the methods need clarification. The sample size justification is internally inconsistent and mixes different rationales. In addition, the study uses convenience sampling from a single center, which limits generalizability and should be more explicitly acknowledged in the interpretation. The manuscript should also clarify the unit of analysis, including how right and left molars were handled and whether bilateral observations were treated independently.

Fifth, the discussion and title should be more restrained. The title emphasizes “clinical applications,” and the discussion makes broad forensic and clinical inferences, but the present study is a single-center exploratory morphometric analysis. The practical implications should therefore be toned down unless supported more directly by the data.

Finally, the manuscript would benefit from editorial revision for clarity and consistency. The use of “sex” and “gender” should be standardized, the keyword “innovation” is not informative, and the data availability statement should be made fully consistent with PLOS requirements, since the manuscript text states that data are available on request.

Overall, the study has potential, but the manuscript needs major revision in statistical reporting, methodological transparency, and interpretation before it is suitable for publication.

Reviewer #4: The manuscript aims to evaluate the dimorphism of the first and second molars for clinical applications. It proposes an elaborate technique based on 3D x, y, and z landmark coordinates. However, numerous aspects require enhancement before being considered for publication.

1. The necessity of dimorphism for clinical application is unclear throughout the introduction and discussion. The introduction lacks a definitive application that would substantiate the objectives as a scientific need to be addressed by this research. What is the impetus for your inquiry?

2. Moreover, I recognize that in forensic sciences, this would be pertinent; however, it is not referenced in the introduction, only cited at the conclusion of the debate.

3. Significant emphasis is placed on the embryological and genetic causes of diversity; nevertheless, minimal implementation of this knowledge occurs in practice.

4. The introduction has extensive sections of text lacking citations. In many instances, three or four sentences conclude with a citation, although it remains unclear if all the preceding content belongs to a singular citation. The authors should provide citations for every assertions, even if they are reiterated.

5. The objectives are lengthy and intertwined with the hypothesis explanation. This outlines the objectives and subsequently the aims of the research. It is imperative that the objectives remain prominent throughout the discussion and conclusions.

6. Kindly adhere to the Strobe statement sequence for the elucidation of methodology and outcomes. Commence with ethical considerations, followed by setting and sampling. The document begins with the sample size calculation, followed by an explanation of the requirements for participant recruitment.

7. The approaches must establish explicit inclusion criteria. Dental conditions, the absence of syndromes, and the analyzed population (specifically, why were only individuals with historical roots in this community selected, considering this may introduce a risk of bias?)

8. The age range serves as another illustration. It appears to involve minors, as shown by the acceptance of consent from legal guardians. Given that it is avoidable, why involve minor volunteers if the research could be conducted exclusively with legal age individuals?

9. On page 7, line 160, substitute the phrase “convenience sampling upon their arrival” to eliminate ambiguity, as “convenience sampling” typically refers to a participant selection based on convenience or availability rather than calculation, as employed in this research.

10. A critical aspect of the methodologies is the explicit delineation of the training process to guarantee reproducibility. The analysis must encompass both intra- and interobserver assessments to corroborate the findings, given that the study addresses a limited range of variance (sometimes within a 1mm threshold).

11. A more comprehensive description of the statistical analysis should be provided in the techniques section.

12. The results lack descriptive statistics, limiting comparisons of tooth size and shape with existing or future investigations. The verification of dispersion normalcy must be conducted for each measurement set.

13. It also failed to present some results, including the R², Spearman correlation, and other analyses. A confusion matrix can convert the results into percentages of specificity and sensitivity of the method. Additionally, AUC, collinearity analysis, VIFF, and other statistical components of logistic regression could enrich the results.

14. In-depth application analysis. If a regression analysis was conducted, why not provide an equation or methodology for sex classification based on your findings?

15. The discussion excessively focuses on the genetic factors and embryological origins of diversity, rather than addressing the practical implications of this discovery.

16. Minimal comparisons are made with other studies, and numerous references in the debate predate the threshold of 2020.

Reviewer #5: The geomorphometric dental studies presented in this article have a broad applicability. Methods that allow identification of sex or account for sex-related differences in tooth development can be useful for odontologists, forensic scientists, and bioarchaeologists when it is not possible to determine biological sex using conventional methods.

The submitted article meets the requirements for a scientific publication. It is written in fluent language; the problem is clearly stated; appropriate statistical methods are used; the results are presented clearly; and the tables and figures are informative. The discussion is thorough, and the conclusions are consistent with the results.

However, the topic of the article is sexual dimorphism in the size and shape of molars, which is also stated as the aim of the study. Nevertheless, this main topic is not reflected in the title. Although sexual dimorphism is mentioned in the short title, emphasizing it in the main title would help readers better grasp the article's main issue.

As for the sample size calculation, the determination is based on the publication by Abe K et al. (1966). Why was this particular study selected?

The following remarks are more of an editorial nature:

•In general, the revision of the article by a native English speaker is recommended.

•In cases when a reference or table number is cited at the end of a sentence, the dot is placed before the parentheses. Below are a few examples:

Line 87: “These changes are most easily observed in human molars. [2]”

Line 174: “…using slicermorph software (https://github.com/SlicerMorph, Seattle, USA). [15] (Table 1, Figure 1,2)”

This style of formatting makes the text difficult to read and should be changed to a more common style where the period is placed after the parentheses, unless such a style is specified in the journal’s requirements.

•The use of articles when referring to “the first molar” and “the second molar” needs to be checked. Such definite articles are missing not once in the article.

•When presenting the list of landmarks in the Results section, it is recommended to separate the numbers with spaces: e.g., change “landmarks 11,27” to “landmarks 11, 27” (line 2016).

•In two paragraphs (lines 113–122 and 175–176), the text is written in a smaller font than in the rest of the article.

•The p-values in the article are presented in three different formats: “P value 0.01” (lines 169, 246, 247), “p=0.0012” (line 200), and “p<0.001” (line 201). The presentation of the p-values needs to be standardized.

•Line 133: change to “…the required sample size in each group...”

•Line 142: change to “…resulting in a total of 120 individuals.”

•Lines 196 and 348: the term “gender” refers to a social construct, and should be changed to the biological term “sex”.

•Line 205: change to “…discriminating between the sexes...”

•Line 217: change to “..noted was that the occlusal table…”

•Line 234: the mentioned authors (Butler’s field model (1939) and Osborn’s Clone model (1978)) are not mentioned in the References.

•Line 242: change to “…size is larger than in the group...”

•Lines 248-249: clarify the sentence “The significant difference in the second molar is predominantly attributed due to extent of development the hypocone (distopalatal cusp) development.”

•Line 262: change to “...systems like functional, developmental, and/or genetic factors…”

•Line 284: change to “…to our results for the first molar.”

•Line 309: change to “…multiple factors that influence the shape…”

•Line 316: change to “...to discriminate between sexes in the modern human.”

•Line 343: clarify the sentence “To further understand the genetic basis of tooth morphogenesis within the population, more study is required…”

•Line 359: change to “...from the institutional ethics…”

•Correct the use of capital letters and articles in Tables. For example:

“Landmarks based on cusp” but “Landmarks based on Ridges” or “Buccal crest of curvature on mesiobuccal lobe”, but “Buccal crest of curvature on the Distobuccal lobe” (Table 1)

“Maxillary First molar” (Table 2), but “Maxillary First Molar” (Table 3)

“Prediction accuracy Female” but “Prediction Accuracy Male” (Table 4)

•Table 4: some statistical values in the second column are in bold. Is that necessary?

In summary, the comments provided do not diminish the scientific value of the article. Once the article has been revised, it is definitely suitable for publication.

Reviewer #6: While the topic is relevant, the manuscript contains fundamental methodological and statistical flaws that invalidate the central findings. These are not issues amenable to revision; they would require the study to be substantially redesigned. The key reasons for rejection are outlined below.

The claimed 95% sex classification accuracy is almost certainly an artefact of overfitting. With 84 shape variables and only 120 specimens (60 per sex), the variable-to-sample ratio (~1:1.5) is far below accepted thresholds (1:5–1:10).

The abstract reports UM2 shape as “P<0.01” while Table 2 shows P=0.01 exactly. The inconsistent use of “sex” versus “gender” further reflects a lack of rigour in reporting.

No intra- or inter-observer error analysis is reported for the 28 landmarks (10 of which are geometrically, not anatomically, defined). Without measurement error quantification, it is impossible to determine whether reported shape differences reflect biology or digitisation noise. This is a basic requirement in geometric morphometric studies.

Sample size calculation is based on premolar centroid size data, not molar shape data, making it inappropriate for the study’s primary analyses. Convenience sampling from orthodontic patients introduces selection bias, as these patients may not represent normal dental morphology. The manuscript does not specify which side (left/right) was used or how bilateral data was handled, raising concerns about data independence.

The Discussion is disproportionately long and speculative. Figures are difficult to interpret and lack proper axis labels. The data availability statement contradicts PLOS ONE’s open-data policy. Multiple grammatical errors persist throughout.

**********

6. PLOS authors have the option to publish the peer review history of their article (what does this mean?). If published, this will include your full peer review and any attached files.

If you choose “no”, your identity will remain anonymous but your review may still be made public.

**Do you want your identity to be public for this peer review?**  For information about this choice, including consent withdrawal, please see our Privacy Policy.

Reviewer #1: No

Reviewer #2: **Yes:**  Luisa M. Barin

Reviewer #3: No

Reviewer #4: No

Reviewer #5: No

Reviewer #6: No

**********

[NOTE: If reviewer comments were submitted as an attachment file, they will be attached to this email and accessible via the submission site. Please log into your account, locate the manuscript record, and check for the action link "View Attachments". If this link does not appear, there are no attachment files.]

To ensure your figures meet our technical requirements, please review our figure guidelines: https://journals.plos.org/plosone/s/figures

You may also use PLOS’s free figure tool, NAAS, to help you prepare publication quality figures: https://journals.plos.org/plosone/s/figures#loc-tools-for-figure-preparation.

NAAS will assess whether your figures meet our technical requirements by comparing each figure against our figure specifications.

# Author comment: Author response to Decision Letter 1

**DOI:** 10.1371/journal.pone.0355612.r003

8 May 2026

We thank the editor and all reviewers for their detailed and constructive feedback. The manuscript has been extensively revised to address all concerns raised. Major revisions include improvement in methodological clarity, detailed reporting of statistical analyses, incorporation of reliability assessment, correction of data availability in accordance with journal policy, and alignment of results with conclusions.

The title, abstract, discussion, and conclusions have been revised to avoid overinterpretation and to accurately reflect the study findings. Formatting, language, figure and table presentation, and reference style have been standardized throughout.

A detailed, point-by-point response to all reviewer and editor comments, including corresponding line numbers, has been provided in the uploaded “Response to Reviewers” document.

Reviewer #1

We thank the reviewer for the detailed and critical evaluation. The manuscript has been revised to improve alignment between aims, results, and conclusions. The abstract has been strengthened, statistical reporting standardized, and methodological clarity enhanced. A formal null hypothesis has been incorporated, and overinterpretation of applications has been removed. Language, citation style, and figure/table presentation have been corrected throughout.

Reviewer #2

We thank the reviewer for recognizing the relevance of the study and providing constructive suggestions. The title has been revised to better reflect the study scope. Sample characteristics, including age distribution and handling of bilateral data, have been clarified. Statistical methods and reliability assessment have been expanded, and data availability has been updated with a public repository. The discussion has been strengthened with appropriate references and reduced overstatement.

Reviewer #3

We thank the reviewer for the insightful feedback. The manuscript has been revised to ensure consistency between results and conclusions, particularly clarifying that significant shape differences were observed only for the second molar. The interpretation of discriminant analysis has been moderated, and limitations including lack of cross-validation have been acknowledged. Reliability analysis, methodological transparency, and reporting clarity have been substantially improved.

Reviewer #4

We thank the reviewer for the constructive suggestions. The Introduction has been revised to clarify the rationale and reduce emphasis on direct clinical application. The Methods section has been reorganized according to reporting standards, and inclusion criteria and sampling methodology have been clarified. Statistical methods and reliability assessment have been detailed, and the Discussion has been revised to balance theoretical context with study findings.

• Reviewer #5

We thank the reviewer for the positive evaluation and helpful editorial suggestions. The title has been revised to emphasize sexual dimorphism. Language, formatting, and terminology have been standardized throughout. Table and figure formatting has been corrected, and minor grammatical and stylistic issues have been addressed to improve clarity and consistency.

Reviewer #6

We thank the reviewer for the critical evaluation. The manuscript has been substantially revised to improve methodological transparency and statistical clarity. The interpretation of classification accuracy has been moderated, and limitations including potential overfitting and lack of cross-validation have been explicitly acknowledged. Reliability assessment, sample size clarification, and data handling procedures have been incorporated. The discussion has been refined, and data availability has been aligned with journal policy.

Attachment Submitted filename: Plos One Response to reviewers.docx

# Peer review: Decision Letter 1

**DOI:** 10.1371/journal.pone.0355612.r004

**Authors:** Andrej Kielbassa

20 May 2026

PONE-D-25-59851R1

Geometric morphometric analysis of maxillary molar sexual dimorphism: a cross-sectional study

PLOS One

Dear Dr. Ahmed,

Thank you for submitting your manuscript to PLOS ONE. After careful consideration, we feel that it has merit but does not fully meet PLOS ONE’s publication criteria as it currently stands. Therefore, we invite you to submit a revised version of the manuscript that addresses the points raised during the review process.

Please submit your revised manuscript by Jul 04 2026 11:59PM. If you will need more time than this to complete your revisions, please reply to this message or contact the journal office at plosone@plos.org. When you're ready to submit your revision, log on to https://www.editorialmanager.com/pone/ and select the 'Submissions Needing Revision' folder to locate your manuscript file.

Please include the following items when submitting your revised manuscript:

- A letter that responds to each point raised by the academic editor and reviewer(s). You should upload this letter as a separate file labeled 'Response to Reviewers'.
- A marked-up copy of your manuscript that highlights changes made to the original version. You should upload this as a separate file labeled 'Revised Manuscript with Track Changes'.
- An unmarked version of your revised paper without tracked changes. You should upload this as a separate file labeled 'Manuscript'.

If you would like to make changes to your financial disclosure, please include your updated statement in your cover letter. Guidelines for resubmitting your figure files are available below the reviewer comments at the end of this letter.

If applicable, we recommend that you deposit your laboratory protocols in protocols.io to enhance the reproducibility of your results. Protocols.io assigns your protocol its own identifier (DOI) so that it can be cited independently in the future. For instructions see: https://journals.plos.org/plosone/s/submission-guidelines#loc-laboratory-protocols. Additionally, PLOS ONE offers an option for publishing peer-reviewed Lab Protocol articles, which describe protocols hosted on protocols.io. Read more information on sharing protocols at https://plos.org/protocols?utm_medium=editorial-email&utm_source=authorletters&utm_campaign=protocols.

As the corresponding author, your ORCID iD is verified in the submission system and will appear in the published article. PLOS supports the use of ORCID, and we encourage all coauthors to register for an ORCID iD and use it as well. Please encourage your coauthors to verify their ORCID iD within the submission system before final acceptance, as unverified ORCID iDs will not appear in the published article. *Only*  the individual author can complete the verification step; PLOS staff *cannot*  verify ORCID iDs on behalf of authors.

We look forward to receiving your revised manuscript.

Kind regards,

Andrej M Kielbassa

Academic Editor

PLOS One

Journal Requirements:

1. If the reviewer comments include a recommendation to cite specific previously published works, please review and evaluate these publications to determine whether they are relevant and should be cited. There is no requirement to cite these works unless the editor has indicated otherwise.

Additional Editor Comments:

Please note that after having double checked your revised and re-submitted draft (see Reviewer #1), I have invited our external reviewers, to complete this review process.

[Note: HTML markup is below. Please do not edit.]

Reviewer's Responses to Questions

**Comments to the Author**

1. If the authors have adequately addressed your comments raised in a previous round of review and you feel that this manuscript is now acceptable for publication, you may indicate that here to bypass the “Comments to the Author” section, enter your conflict of interest statement in the “Confidential to Editor” section, and submit your "Accept" recommendation.

Reviewer #1: All comments have been addressed

Reviewer #2: All comments have been addressed

Reviewer #3: (No Response)

Reviewer #5: All comments have been addressed

**********

2. Is the manuscript technically sound, and do the data support the conclusions?

The manuscript must describe a technically sound piece of scientific research with data that supports the conclusions. Experiments must have been conducted rigorously, with appropriate controls, replication, and sample sizes. The conclusions must be drawn appropriately based on the data presented.

Reviewer #1: Yes

Reviewer #2: Yes

Reviewer #3: Partly

Reviewer #5: Yes

**********

3. Has the statistical analysis been performed appropriately and rigorously?

Reviewer #1: Yes

Reviewer #2: Yes

Reviewer #3: No

Reviewer #5: Yes

**********

4. Have the authors made all data underlying the findings in their manuscript fully available?

The PLOS Data policy requires authors to make all data underlying the findings described in their manuscript fully available without restriction, with rare exception (please refer to the Data Availability Statement in the manuscript PDF file). The data should be provided as part of the manuscript or its supporting information, or deposited to a public repository. For example, in addition to summary statistics, the data points behind means, medians and variance measures should be available. If there are restrictions on publicly sharing data—e.g. participant privacy or use of data from a third party—those must be specified.

Reviewer #1: No

Reviewer #2: Yes

Reviewer #3: Yes

Reviewer #5: Yes

**********

5. Is the manuscript presented in an intelligible fashion and written in standard English?

PLOS ONE does not copyedit accepted manuscripts, so the language in submitted articles must be clear, correct, and unambiguous. Any typographical or grammatical errors should be corrected at revision, so please note any specific errors here.

Reviewer #1: Yes

Reviewer #2: Yes

Reviewer #3: Yes

Reviewer #5: No

**********

6. Review Comments to the Author

Please use the space provided to explain your answers to the questions above. You may also include additional comments for the author, including concerns about dual publication, research ethics, or publication ethics. (Please upload your review as an attachment if it exceeds 20,000 characters)

Reviewer #1: The previous Reviewers' comments have considerably improved this revised and re-submitted draft. All comments have been addressed, and all recommendations have been followed. In total, the Co-Authors responses would seem satisfying, and this manuscript is considered ready to proceed.

Reviewer #2: The manuscript shows significant improvement compared to the previous version, particularly regarding the alignment between title, objective, methodology, results, and conclusion. The focus on shape analysis using geometric morphometric analysis has become more consistent throughout the manuscript, and there has been an important improvement in the cautious interpretation of the findings, especially after inclusion of the limitations related to the absence of cross-validation and the overlap observed in the PCA. Nevertheless, some methodological and interpretative weaknesses still remain, particularly regarding discriminant function analysis (DFA), potential overfitting and lack of validation, biological inferences extending beyond the data actually produced, the excessively narrative character of the discussion, and a conclusion that remains slightly assertive in some aspects.

Introduction:

- Some sections remain excessively extensive, particularly those related to genetic, chromosomal, and dental developmental mechanisms, which were not directly evaluated in the study;

- The first paragraph still appears overly focused on digital scanning technologies and prosthetic/aesthetic applications, topics that do not directly represent the primary focus of the research;

- There is slight redundancy in sections discussing “shape versus size”;

- Consolidating references at the end of conceptual blocks could improve readability and avoid excessive citation repetition;

Methodology:

- The expression “obtained prospectively from orthodontic patient records” may create ambiguity between prospective data collection and retrospective analysis of existing clinical records. Clarifying the temporal process of data acquisition could improve methodological transparency;

- The justification for population homogeneity based on individuals from the same region could be presented more objectively and scientifically;

- Convenience sampling was appropriately declared; however, its potential biases could be acknowledged more explicitly;

- Sample size calculation was based on linear odontometric measurements, whereas the primary outcome of the study involves multivariate geometric shape analysis. Better methodological alignment or statistical justification could strengthen this section;

- Information regarding landmark reproducibility remains absent, including intra/interexaminer error assessment, calibration, and landmark reliability evaluation;

- There is some redundancy between the ethics and consent sections.

Results:

- Interpretation of the high discriminant accuracy (~95%) still requires caution, particularly considering:

lack of statistical significance in part of the results;

- overlap between groups in the PCA;

- absence of cross-validation;

- The absence of cross-validation represents an important methodological limitation and likely contributes to overestimation of classification accuracy;

- Some anatomical interpretations related to landmark displacement remain excessively descriptive and could be synthesized to improve objectivity;

- The results section could be slightly condensed, particularly regarding detailed anatomical descriptions of landmarks.

Discussion:

- The discussion remains excessively extensive and narrative in certain sections, particularly in explanations involving genetic mechanisms, developmental dental theories, and molecular influences, aspects that were not directly evaluated in the study;

- Some interpretations still appear overly inferential in relation to the actual data obtained, especially regarding discriminative power, biological mechanisms, and possible clinical/forensic applications;

- The high discriminant accuracy observed should continue to be interpreted cautiously, considering the absence of cross-validation, the overlap observed in the PCA, and the lack of statistical significance in part of the results.

Limitations related to landmark reproducibility, intra/interexaminer reliability assessment, and external validation of discriminant models remain insufficiently addressed;

- Some sections appear peripheral to the primary objective of the study and could be condensed to improve objectivity and readability of the discussion.

Conclusion:

- The conclusion could briefly mention that the findings specifically refer to the studied population, reinforcing the limitation of generalizability already discussed previously and maintaining better coherence with the methodological limitations presented in the discussion.

General Suggestions:

Consider reporting more explicitly the landmark validation/reproducibility procedures in the main text (e.g., intraexaminer error, repeated landmark placement, or ICC), as this is particularly relevant in geometric morphometric studies;

Interpretation of the discriminant function analysis (DFA) could be presented more conservatively, emphasizing that high classification accuracy does not necessarily imply high generalizable discriminative capability, especially in the absence of external validation or cross-validation;

The discussion could be simplified by reducing lengthy theoretical sections related to biological/genetic mechanisms not directly evaluated in the study, prioritizing greater focus on findings actually produced by the morphometric analysis;

Inclusion of a summarized methodological flowchart or analytical pipeline could improve clarity and reproducibility of the study, especially for readers less familiar with geometric morphometric analysis;

A final revision of scientific language and textual fluency by a specialized editing service or native English reviewer is recommended to improve readability, objectivity, and overall manuscript quality.

Grammatical:

Lines 62–63 (“holds immense potential” / “assisting prosthetic dentists in fabricating dental tooth surfaces”), line 66 (“One well-known example”), lines 82–84 (“derived from the ectoderm interact with the dental papilla”), line 109 (“Presently, majority of the research”), lines 109–110 (“focuses on size rather than shape of biological entities”), lines 112–113 (“foundational basis for future applications”), line 127 (“obtained prospectively from orthodontic patient records”), line 134 (“A detailed history of the demographics including the domicile/birthplace”), line 136 (“born and brought up”), line 159 (“improve robustness”), line 162 (“ensuring stability of the analysis”), lines 175–176 (“provided a comprehensive framework”), line 239 (“variation between the sex”), line 245 (“significant association of size dependent shape change”), line 252 (“for prediction of the shape”), line 254 (“which indicate the mean difference”), and lines 301–302 (“female teeth also were seen to show”) could be revised regarding methodological clarity, scientific objectivity, academic fluency, and grammatical/stylistic adequacy.

Reviewer #3: This revised version is clearly improved. The title is now more appropriate, the conclusions are better aligned with the reported findings, landmark reliability has been added, bilateral handling is described, and the data availability statement is now repository-based. The core result is also presented more accurately: there is no significant sex-related shape difference for UM1, whereas UM2 shows a significant shape difference; allometry is significant in both teeth and larger in UM2.

That said, I do not think the manuscript is ready for acceptance yet. The main remaining issue is that the paper still gives too much weight to the discriminant/classification analysis. The abstract states that discriminant function analysis showed around 95% classification accuracy, but the methods make clear that this was calculated on the original dataset, without cross-validation, and the results also show substantial overlap in PCA and non-significant DFA p values. In this form, the classification result is not robust evidence of discriminatory performance and should not be highlighted in the abstract or interpreted as demonstrating practical sex-classification utility. It should be clearly framed as an apparent, in-sample result only.

A second important issue is the treatment of bilateral data. The authors state that both right and left maxillary molars were included and that averaged landmark coordinates were used per individual. However, the manuscript does not explain whether left teeth were mirrored/reflected into a common orientation before averaging. In a 3D geometric morphometric workflow, this is methodologically important. Without a clear statement on mirroring and alignment of antimers, the averaging approach is difficult to evaluate. This point requires explicit clarification, and if mirroring was not performed, the analysis may need correction.

The sample-size section is improved compared with the earlier version, but it still does not fully match the primary analysis. The calculation is based on previously reported linear mesiodistal tooth dimensions, whereas the study’s main endpoint is multivariate 3D shape. That may be acceptable as a pragmatic proxy in an exploratory study, but it should be presented as such and not as a firm justification that the sample is adequate for geometric morphometric inference. The sentence implying that the sample size “ensured stability of the analysis” is stronger than the methods support.

The discussion remains better than before, but it still contains some overinterpretation and speculation. In particular, the statement that the second molar “may be used with sufficient power to discriminate between sexes in the modern human” is not supported by the current analytical framework, especially given the lack of cross-validation and the overlap in PCA. Likewise, the broader orthodontic and forensic implications should be phrased much more cautiously unless the authors explicitly state that these are possible future directions rather than conclusions of the present study. The developmental/genetic explanations are plausible, but they remain interpretive rather than directly tested here and should be framed accordingly.

The added reliability analysis is a useful improvement, but the reporting is still somewhat incomplete. The manuscript reports ICC values from 0.982 to 1.000 and states that digitization error accounted for less than 10% of variance, but it does not fully explain whether the ICCs were calculated per landmark, per coordinate set, or as a global summary. The nonparametric ANOVA-based digitization-error analysis also needs slightly clearer description so readers can understand exactly what was repeated and how error was partitioned.

There are also some smaller issues of wording and presentation. The phrasing “pretreatment dental casts were obtained prospectively from orthodontic patient records” is awkward and should be clarified. The age distribution is now reported, which is helpful, but the modest age difference between males and females should at least be acknowledged, even if the authors believe it is unlikely to affect crown morphology materially in this young sample. The language overall is much improved, but the manuscript would still benefit from another careful stylistic edit.

In summary, this revision is substantially better and closer to publishable form. The descriptive morphometric finding for UM2 is potentially publishable. However, the bilateral-data workflow must be clarified, the classification analysis must be substantially toned down, and the discussion should be further restrained. If those points are addressed carefully, I think the manuscript could become suitable for publication.

Reviewer #5: The authors have taken my comments into account. Following the revisions, the quality of the article has improved significantly.

I would recommend checking the manuscript once more for still left proofreading errors: for example, removing text highlighting (lines 48, 49, 73), standardize the font size (lines 184, 306), add missing periods at the end of sentences (lines 215, 442), separate words (lines 167, 170), and check the use of capital letters in Table 1 (landmarks 13, 15, 16).

**********

7. PLOS authors have the option to publish the peer review history of their article (what does this mean?). If published, this will include your full peer review and any attached files.

If you choose “no”, your identity will remain anonymous but your review may still be made public.

**Do you want your identity to be public for this peer review?**  For information about this choice, including consent withdrawal, please see our Privacy Policy.

Reviewer #1: No

Reviewer #2: No

Reviewer #3: No

Reviewer #5: No

**********

[NOTE: If reviewer comments were submitted as an attachment file, they will be attached to this email and accessible via the submission site. Please log into your account, locate the manuscript record, and check for the action link "View Attachments". If this link does not appear, there are no attachment files.]

To ensure your figures meet our technical requirements, please review our figure guidelines: https://journals.plos.org/plosone/s/figures

You may also use PLOS’s free figure tool, NAAS, to help you prepare publication quality figures: https://journals.plos.org/plosone/s/figures#loc-tools-for-figure-preparation.

NAAS will assess whether your figures meet our technical requirements by comparing each figure against our figure specifications.

# Author comment: Author response to Decision Letter 2

**DOI:** 10.1371/journal.pone.0355612.r005

15 Jun 2026

Dear Reviewers,

We sincerely thank all reviewers for their careful evaluation of our manuscript and for the constructive comments and suggestions provided during this round of review. We greatly appreciate the time and effort invested in improving the quality, clarity, and scientific rigor of our work.

### Reviewer #1

We are grateful for the reviewer’s positive assessment and recognition that the manuscript has improved substantially following revision. In response to the remaining comments, we further refined the Introduction by reducing emphasis on digital scanning technologies, condensing sections related to developmental and genetic mechanisms, minimizing redundancy regarding shape-versus-size concepts, and improving citation organization. Additional methodological clarifications and language revisions were also incorporated throughout the manuscript.

### Reviewer #2

We thank the reviewer for the detailed methodological and interpretative feedback. In response, we improved methodological transparency by clarifying the source of study materials, population-selection criteria, convenience sampling limitations, and sample-size justification. Landmark reproducibility assessment, observer calibration procedures, reliability testing, and digitization-error analyses have been more explicitly described. We also substantially moderated the interpretation of discriminant function analysis, emphasized the absence of cross-validation, reduced overinterpretation of classification accuracy, condensed portions of the Results and Discussion, and revised the Conclusion to better reflect the population-specific nature of the findings.

### Reviewer #3

We appreciate the reviewer’s recognition of the improvements made during the previous revision as well as the additional recommendations provided. In response, we clarified the bilateral geometric morphometric workflow by explicitly describing the mirroring and averaging procedures used for antimeric teeth. We expanded the reporting of landmark reproducibility assessment, clarified that ICC values represent global reliability estimates, and provided additional details regarding digitization-error analysis. The Abstract, Results, Discussion, and Conclusion were revised to further reduce emphasis on classification performance and to clearly distinguish observed findings from literature-based interpretations. Developmental, genetic, orthodontic, and forensic implications were reframed as contextual interpretations or future research directions rather than direct conclusions of the study.

### Reviewer #5

We thank the reviewer for the positive assessment of the revised manuscript. In response to the remaining editorial comments, we carefully proofread the manuscript and corrected formatting inconsistencies, typographical errors, punctuation issues, spacing errors, capitalization inconsistencies, and other minor language-related concerns. The manuscript has also undergone an additional review for readability, consistency, and overall presentation.

A detailed point-by-point response addressing every reviewer comment is provided in the accompanying “Response to Reviewers” document.

We sincerely appreciate the reviewers’ insightful comments and believe that the revisions have substantially strengthened the manuscript. We hope that the revised version satisfactorily addresses all concerns and is now suitable for publication.

With sincere thanks,

The Authors

Attachment Submitted filename: Plos_One_Response_to_reviewers_auresp_2.docx

# Peer review: Decision Letter 2

**DOI:** 10.1371/journal.pone.0355612.r006

**Authors:** Andrej Kielbassa

21 Jun 2026

PONE-D-25-59851R2

Geometric morphometric analysis of maxillary molar sexual dimorphism: a cross-sectional study

PLOS One

Dear Dr. Ahmed,

Thank you for submitting your manuscript to PLOS ONE. After careful consideration, we feel that it has merit but does not fully meet PLOS ONE’s publication criteria as it currently stands. Therefore, we invite you to submit a revised version of the manuscript that addresses the points raised during the review process.

Please submit your revised manuscript by Aug 05 2026 11:59PM. If you will need more time than this to complete your revisions, please reply to this message or contact the journal office at plosone@plos.org. When you're ready to submit your revision, log on to https://www.editorialmanager.com/pone/ and select the 'Submissions Needing Revision' folder to locate your manuscript file.

Please include the following items when submitting your revised manuscript:

- A letter that responds to each point raised by the academic editor and reviewer(s). You should upload this letter as a separate file labeled 'Response to Reviewers'.
- A marked-up copy of your manuscript that highlights changes made to the original version. You should upload this as a separate file labeled 'Revised Manuscript with Track Changes'.
- An unmarked version of your revised paper without tracked changes. You should upload this as a separate file labeled 'Manuscript'.

If you would like to make changes to your financial disclosure, please include your updated statement in your cover letter. Guidelines for resubmitting your figure files are available below the reviewer comments at the end of this letter.

If applicable, we recommend that you deposit your laboratory protocols in protocols.io to enhance the reproducibility of your results. Protocols.io assigns your protocol its own identifier (DOI) so that it can be cited independently in the future. For instructions see: https://journals.plos.org/plosone/s/submission-guidelines#loc-laboratory-protocols. Additionally, PLOS ONE offers an option for publishing peer-reviewed Lab Protocol articles, which describe protocols hosted on protocols.io. Read more information on sharing protocols at https://plos.org/protocols?utm_medium=editorial-email&utm_source=authorletters&utm_campaign=protocols.

As the corresponding author, your ORCID iD is verified in the submission system and will appear in the published article. PLOS supports the use of ORCID, and we encourage all coauthors to register for an ORCID iD and use it as well. Please encourage your coauthors to verify their ORCID iD within the submission system before final acceptance, as unverified ORCID iDs will not appear in the published article. *Only*  the individual author can complete the verification step; PLOS staff *cannot*  verify ORCID iDs on behalf of authors.

We look forward to receiving your revised manuscript.

Kind regards,

Andrej M Kielbassa

Academic Editor

PLOS One

Journal Requirements:

If the reviewer comments include a recommendation to cite specific previously published works, please review and evaluate these publications to determine whether they are relevant and should be cited. There is no requirement to cite these works unless the editor has indicated otherwise.

Please review your reference list to ensure that it is complete and correct. If you have cited papers that have been retracted, please include the rationale for doing so in the manuscript text, or remove these references and replace them with relevant current references. Any changes to the reference list should be mentioned in the rebuttal letter that accompanies your revised manuscript. If you need to cite a retracted article, indicate the article’s retracted status in the References list and also include a citation and full reference for the retraction notice.

Additional Editor Comments:

Please note that I have double checked your revised and re-submitted version of your draft (see R #1). Remember that several minor shortcomings would call for further improvements.

[Note: HTML markup is below. Please do not edit.]

Reviewers' comments:

Reviewer's Responses to Questions

**Comments to the Author**

1. If the authors have adequately addressed your comments raised in a previous round of review and you feel that this manuscript is now acceptable for publication, you may indicate that here to bypass the “Comments to the Author” section, enter your conflict of interest statement in the “Confidential to Editor” section, and submit your "Accept" recommendation.

Reviewer #1: All comments have been addressed

Reviewer #3: All comments have been addressed

Reviewer #5: All comments have been addressed

**********

2. Is the manuscript technically sound, and do the data support the conclusions?

The manuscript must describe a technically sound piece of scientific research with data that supports the conclusions. Experiments must have been conducted rigorously, with appropriate controls, replication, and sample sizes. The conclusions must be drawn appropriately based on the data presented.

Reviewer #1: No

Reviewer #3: Partly

Reviewer #5: Yes

**********

3. Has the statistical analysis been performed appropriately and rigorously?

Reviewer #1: Yes

Reviewer #3: Yes

Reviewer #5: Yes

**********

4. Have the authors made all data underlying the findings in their manuscript fully available?

The PLOS Data policy requires authors to make all data underlying the findings described in their manuscript fully available without restriction, with rare exception (please refer to the Data Availability Statement in the manuscript PDF file). The data should be provided as part of the manuscript or its supporting information, or deposited to a public repository. For example, in addition to summary statistics, the data points behind means, medians and variance measures should be available. If there are restrictions on publicly sharing data—e.g. participant privacy or use of data from a third party—those must be specified.

Reviewer #1: Yes

Reviewer #3: Yes

Reviewer #5: Yes

**********

5. Is the manuscript presented in an intelligible fashion and written in standard English?

PLOS ONE does not copyedit accepted manuscripts, so the language in submitted articles must be clear, correct, and unambiguous. Any typographical or grammatical errors should be corrected at revision, so please note any specific errors here.

Reviewer #1: Yes

Reviewer #3: Yes

Reviewer #5: Yes

**********

6. Review Comments to the Author

Please use the space provided to explain your answers to the questions above. You may also include additional comments for the author, including concerns about dual publication, research ethics, or publication ethics. (Please upload your review as an attachment if it exceeds 20,000 characters)

Reviewer #1: With the help of the Reviewers, this submitted draft could be considerably improved. Notwithstanding, several further revisions would seem mandatory, please see below.

Abstract

- "(...), explained by the concept in literature of farther distance from the primary field of influence and genetic associations." This is not considered a conclusion from your study.

- With your Conclusions, do not simply repeat your outcome. Do not speculate. Stick exclusively to your aims, and provide generalizing responses.

Intro

- "Tooth size is influenced by a variety of (...)." Remember that one sentence does not constitute one paragraph. Provide digestible units.

Meths

- "(...) and with the 1964 Helsinki Declaration and its later amendments or comparable ethical standards." Reference missing.

- "This study was not pre125 registered as it was an observational cross-sectional study using existing clinical records." Remember that "existing clinical records" would not seem a sound reason to NOT pre-register. The latter refers to several other reasons. Please revise carefully

- "(...) cannot be assumed.." - revise for typos.

- Same with "(...) was not analysed separately. ." Revise carefully.

- Please revise carefully for uniform expressions. Compare "distolingual/mesiolingual" and "distopalatal/mesiopalatal".

- "The null hypothesis tested was that there is no significant difference in shape or size of maxillary molars between males and females." H0 already has been provided with the Intro section. Why do you repeat this H0 here?

Results

- "mean age of 18.62 years (SD = 2.49)" - this would seem spuriously accurate. One digit would seem sufficient. Revise carefully.

- Again, revise for sound full stop use, see "(...) (p ≤ 0.001). (Table 3) ."

- Same with "(...) based on shape variables. [3]." Revise carefully.

- Why do you stick to "≤"? See "(p ≤ 0.001)".

Disc

- "The null hypothesis stated that there is no significant difference in the shape or size of maxillary permanent molars between males and females." See comments on repetition of H0 given above, and revise carefully.

- Again, revise for uniform formatting, see "p = 0.01" and "p= 0.150".

- "The shape of the molar has been correlated with craniofacial complexes by Polychronis and Halazonetis (2014)." Reference number is missing.

- Again, please compare "distopalatal cusp" and "mesiolingual cusp". Revise carefully for uniform terms and descriptions.

- Again, revise for typos, see "(...) and dental morphology. [32,34,35]."

- Same with "area-based and Procrustes-based approaches".

- Revise for uniform citation style. See "Morita W et al. (2016)", and compare to "Popovici et al (2022)". Revise carefully (as a general recommendation, please reduce Authors' names with your text; instead, stick to your main thoughts).

- "This is consistent with the study done by Morita W et al. (2016), who studied the metameric variation of human molars." Again, reference number is missing.

Figs

- Figures as attached to this manuscript would not seem reproducible. Please double check, and ensure to provide high-quality images.

Reviewer #3: Recommendation: Minor Revision

This version is substantially improved and much closer to publication. The main conclusions are now better aligned with the data: the manuscript clearly states that shape differences were significant only for UM2, not UM1; the abstract and discussion now acknowledge that the classification findings should be interpreted cautiously because no cross-validation was performed; the bilateral workflow is clarified by stating that left teeth were mirrored before averaging; the reliability section is more complete; and the sample-size justification is presented more honestly as a pragmatic proxy based on prior linear odontometric data.

The remaining issues are now mostly about emphasis and presentation rather than fundamental methodological flaws. The main one is that the manuscript still gives quite a lot of prominence to the discriminant analysis: the Results section still reports ~95% apparent accuracy, Table 4 still centers those numbers, and Figure 4 still visually highlights “Accuracy 95%,” even though the manuscript also states that these are in-sample results without cross-validation and that PCA shows substantial overlap with non-significant DFA p values. I suggest further toning this down in the main text, or moving part of it to supplementary material (Figure 4), so readers do not overinterpret exploratory separation as validated classification performance.

The discussion is improved and is generally more restrained, but the conclusion still edges slightly toward application. The final sentence says that the developmental and applied implications warrant future investigation for clinical applications in orthodontics and forensic odontology. That is acceptable only if framed strictly as future work; it should not read as an implication already supported by this study. I would recommend softening this final sentence further.

There are also some residual editorial issues. The manuscript is much cleaner than before, but minor redundancies, small grammatical problems, and a few awkward sentences remain, especially in the Introduction and Discussion. These are readily fixable in a final revision.

Overall, I think the authors have addressed the main scientific concerns from prior rounds. At this stage, I would support publication after minor revision, mainly to further de-emphasize the unvalidated classification results, slightly tighten the conclusion, and complete a final language edit.

Reviewer #5: The authors have substantially improved their work. All of my comments have been addressed. The paper is now acceptable for publication.

**********

7. PLOS authors have the option to publish the peer review history of their article (what does this mean?). If published, this will include your full peer review and any attached files.

If you choose “no”, your identity will remain anonymous but your review may still be made public.

**Do you want your identity to be public for this peer review?**  For information about this choice, including consent withdrawal, please see our Privacy Policy.

Reviewer #1: No

Reviewer #3: No

Reviewer #5: No

**********

[NOTE: If reviewer comments were submitted as an attachment file, they will be attached to this email and accessible via the submission site. Please log into your account, locate the manuscript record, and check for the action link "View Attachments". If this link does not appear, there are no attachment files.]

To ensure your figures meet our technical requirements, please review our figure guidelines: https://journals.plos.org/plosone/s/figures

You may also use PLOS’s free figure tool, NAAS, to help you prepare publication quality figures: https://journals.plos.org/plosone/s/figures#loc-tools-for-figure-preparation.

NAAS will assess whether your figures meet our technical requirements by comparing each figure against our figure specifications.

# Author comment: Author response to Decision Letter 3

**DOI:** 10.1371/journal.pone.0355612.r007

13 Jul 2026

Reviewer #1

We sincerely thank Reviewer #1 for the detailed and constructive comments. In response, we revised the abstract and conclusion to avoid speculation and better align with the study objectives, improved the structure of the Introduction, added the appropriate reference for the Declaration of Helsinki, clarified the preregistration statement, removed repetition of the null hypothesis, standardized anatomical terminology and citation style throughout the manuscript, corrected typographical and formatting inconsistencies (including punctuation and p-value reporting), ensured uniform statistical reporting, added missing references, and improved the quality and presentation of the figures. The manuscript was also carefully proofread to ensure consistency and readability throughout.

Reviewer #3

We sincerely thank Reviewer #3 for the positive assessment and valuable suggestions. In response, we further reduced the emphasis on the exploratory discriminant function analysis by revising the Results section, updating Table 4, and modifying Figure 4 to remove the visual emphasis on classification accuracy. The Discussion and Conclusion were further refined to ensure that interpretations remained fully supported by the study findings and that potential orthodontic and forensic applications were presented only as directions for future research. In addition, a comprehensive editorial revision was undertaken to improve language, readability, formatting, and overall presentation throughout the manuscript.

Reviewer #5

We sincerely thank Reviewer #5 for the positive evaluation of our revised manuscript and for confirming that all previous concerns had been satisfactorily addressed. We greatly appreciate the constructive feedback provided throughout the review process, which has helped improve the quality and clarity of the manuscript.

Attachment Submitted filename: Response to reviewer revision 3.docx

# Peer review: Decision Letter 3

**DOI:** 10.1371/journal.pone.0355612.r008

**Authors:** Andrej Kielbassa

23 Jul 2026

Geometric morphometric analysis of maxillary molar sexual dimorphism: a cross-sectional study

PONE-D-25-59851R3

Dear Dr. Ahmed,

We’re pleased to inform you that your manuscript has been judged scientifically suitable for publication and will be formally accepted for publication once it meets all outstanding technical requirements.

Within one week, you’ll receive an e-mail detailing the required amendments. When these have been addressed, you’ll receive a formal acceptance letter and your manuscript will be scheduled for publication.

An invoice will be generated when your article is formally accepted. Please note, if your institution has a publishing partnership with PLOS and your article meets the relevant criteria, all or part of your publication costs will be covered. Please make sure your user information is up-to-date by logging into Editorial Manager at Editorial Manager® and clicking the ‘Update My Information' link at the top of the page. For questions related to billing, please contact billing support.

If your institution or institutions have a press office, please notify them about your upcoming paper to help maximize its impact. If they’ll be preparing press materials, please inform our press team as soon as possible -- no later than 48 hours after receiving the formal acceptance. Your manuscript will remain under strict press embargo until 2 pm Eastern Time on the date of publication. For more information, please contact onepress@plos.org.

Kind regards,

Prof. Dr. Dr. h. c. Andrej M Kielbassa

Academic Editor

PLOS One

Additional Editor Comments (optional):

Please note that the previous Reviewer did not find any time to re-evaluate your revised draft. Consequently, I have double-checked your re-submitted draft (please see Rev # 1).

Best regards,

Andrej Kielbassa

Reviewers' comments:

Reviewer's Responses to Questions

**Comments to the Author**

1. If the authors have adequately addressed your comments raised in a previous round of review and you feel that this manuscript is now acceptable for publication, you may indicate that here to bypass the “Comments to the Author” section, enter your conflict of interest statement in the “Confidential to Editor” section, and submit your "Accept" recommendation.

Reviewer #1: All comments have been addressed

**********

2. Is the manuscript technically sound, and do the data support the conclusions?

The manuscript must describe a technically sound piece of scientific research with data that supports the conclusions. Experiments must have been conducted rigorously, with appropriate controls, replication, and sample sizes. The conclusions must be drawn appropriately based on the data presented.

Reviewer #1: Yes

**********

3. Has the statistical analysis been performed appropriately and rigorously?

Reviewer #1: Yes

**********

4. Have the authors made all data underlying the findings in their manuscript fully available?

The PLOS Data policy requires authors to make all data underlying the findings described in their manuscript fully available without restriction, with rare exception (please refer to the Data Availability Statement in the manuscript PDF file). The data should be provided as part of the manuscript or its supporting information, or deposited to a public repository. For example, in addition to summary statistics, the data points behind means, medians and variance measures should be available. If there are restrictions on publicly sharing data—e.g. participant privacy or use of data from a third party—those must be specified.

Reviewer #1: Yes

**********

5. Is the manuscript presented in an intelligible fashion and written in standard English?

PLOS ONE does not copyedit accepted manuscripts, so the language in submitted articles must be clear, correct, and unambiguous. Any typographical or grammatical errors should be corrected at revision, so please note any specific errors here.

Reviewer #1: Yes

**********

6. Review Comments to the Author

Please use the space provided to explain your answers to the questions above. You may also include additional comments for the author, including concerns about dual publication, research ethics, or publication ethics. (Please upload your review as an attachment if it exceeds 20,000 characters)

Reviewer #1: With the help of the Reviewers, this draft could be improved. Revisions would seem satisfying. This revised and re-submitted draft is considered ready to proceed.

**********

7. PLOS authors have the option to publish the peer review history of their article (what does this mean?). If published, this will include your full peer review and any attached files.

If you choose “no”, your identity will remain anonymous but your review may still be made public.

**Do you want your identity to be public for this peer review?**  For information about this choice, including consent withdrawal, please see our Privacy Policy.

Reviewer #1: No

**********

# Editor report: Acceptance letter

**DOI:** 10.1371/journal.pone.0355612.r009

**Authors:** Andrej Kielbassa

PONE-D-25-59851R3

PLOS One

Dear Dr. Ahmed,

I'm pleased to inform you that your manuscript has been deemed suitable for publication in PLOS One. Congratulations! Your manuscript is now being handed over to our production team.

At this stage, our production department will prepare your paper for publication. This includes ensuring the following:

* All references, tables, and figures are properly cited

* All relevant supporting information is included in the manuscript submission,

* There are no issues that prevent the paper from being properly typeset

You will receive further instructions from the production team, including instructions on how to review your proof when it is ready. Please keep in mind that we are working through a large volume of accepted articles, so please give us a few days to review your paper and let you know the next and final steps.

Lastly, if your institution or institutions have a press office, please let them know about your upcoming paper now to help maximize its impact. If they'll be preparing press materials, please inform our press team within the next 48 hours. Your manuscript will remain under strict press embargo until 2 pm Eastern Time on the date of publication. For more information, please contact onepress@plos.org.

You will receive an invoice from PLOS for your publication fee after your manuscript has reached the completed accept phase. If you receive an email requesting payment before acceptance or for any other service, this may be a phishing scheme. Learn how to identify phishing emails and protect your accounts at https://explore.plos.org/phishing.

If we can help with anything else, please email us at customercare@plos.org.

Thank you for submitting your work to PLOS ONE and supporting open access.

Kind regards,

PLOS ONE Editorial Office Staff

on behalf of

Prof. Dr. med. dent. Dr. h. c. Andrej M Kielbassa

Academic Editor

PLOS One
